# Site-Specific Cleavage of RNAs Derived from the *PIM1* 3′-UTR by a Metal-Free Artificial Ribonuclease

**DOI:** 10.3390/molecules24040807

**Published:** 2019-02-23

**Authors:** Felix Zellmann, Laura Thomas, Ute Scheffer, Roland K. Hartmann, Michael W. Göbel

**Affiliations:** 1 Institute of Organic Chemistry and Chemical Biology, Goethe University Frankfurt, Max-von-Laue-Str. 7, D-60438 Frankfurt am Main, Germany; Felix.Zellmann@gmx.net (F.Z.); U.Scheffer@chemie.uni-frankfurt.de (U.S.); 2 Institute of Pharmaceutical Chemistry, Philipps University Marburg, Marbacher Weg 6-10, D-35032 Marburg, Germany; laura.thomas@pharmazie.uni-marburg.de (L.T.); roland.hartmann@staff.uni-marburg.de (R.K.H.)

**Keywords:** 2-aminobenzimidazole, cleavage of large RNA molecules, cleavage site selection, DNA/LNA mixmers, dye labeling, guanidine analogs, oligonucleotides, specificity of cleavage

## Abstract

Oligonucleotide conjugates of tris(2-aminobenzimidazole) have been reported previously to cleave complementary RNA strands with high levels of sequence and site specificity. The RNA substrates used in these studies were oligonucleotides not longer than 29-mers. Here we show that ~150–400-mer model transcripts derived from the 3′-untranslated region of the *PIM1* mRNA reacted with rates and specificities comparable to those of short oligonucleotide substrates. The replacement of DNA by DNA/LNA mixmers further increased the cleavage rate. Tris(2-aminobenzimidazoles) were designed to interact with phosphates and phosphate esters. A cell, however, contains large amounts of phosphorylated species that may cause competitive inhibition of RNA cleavage. It is thus important to note that no loss in reaction rates was observed in phosphate buffer. This opens the way to in-cell applications for this type of artificial nuclease. Furthermore, we disclose a new synthetic method giving access to tris(2-aminobenzimidazoles) in multigram amounts.

## 1. Introduction

Synthetic nucleases used to cleave RNA in a sequence-specific manner are normally conjugates of a catalytic subunit and an oligonucleotide part required for substrate recognition via Watson-Crick base pairing [1,2,3,4,5,6]. Complexes of lanthanide ions have been most widely used as RNA cleavers [7,8,9,10]. However, even higher reaction rates have been observed with a Cu^2+^ phenanthroline complex attached to peptide nucleic acids (PNA) [11]. We developed tris(2-aminobenzimidazoles) as a new class of metal-free RNA hydrolyzing agents. When attached to DNA [12] or PNA oligonucleotides [13,14], they hybridize with complementary RNA strands and cut their substrates with half-lives in the range of 10 to 20 h. If the catalyst is positioned at an internal position rather than at the end of the oligonucleotides, product inhibition can be prevented, and multiple substrate turnover occurs [14]. Assays for artificial ribonucleases, including our own work, typically utilize short end-labeled RNAs as targets. A notable exception has been the site-specific cleavage of long transcripts encoding the proto-oncogenic protein kinase c-Raf. A lanthanide complex attached to an oligonucleotide was shown to cleave 60–70% of the substrate at the predicted site within 4 h at 37 °C [15]. PIM1 is another proto-oncogenic serine/threonine kinase that is overexpressed in many types of human cancers and is thus of interest as a drug target [16]. Its mRNA consists of ~2800 nucleotides [17] and includes a long 3′-untranslated region (3′-UTR, ~1400 nt) that harbors regulatory sequences such as an A/U-rich element and a binding site for miR-33a [18]. In addition, the region from position 1491 to 1509 (numbering according to the *PIM1* mRNA sequence [17]) has been shown to be accessible to siRNA-induced degradation [18]. Here, we describe the cleavage kinetics of model RNAs and of transcripts that contain portions of the *PIM1* 3′-UTR. The RNA segments were selected such that their predicted 2D structure was equal or very similar to that predicted for the entire 3′-UTR. In addition to fluorescent labeling of target RNAs, we also analyzed 5′-[^32^P]-end-labeled RNA substrates to avoid any alteration of the RNA’s chemical nature, which could affect the kinetics of the reaction.

## 2. Results

**Scheme 1:**
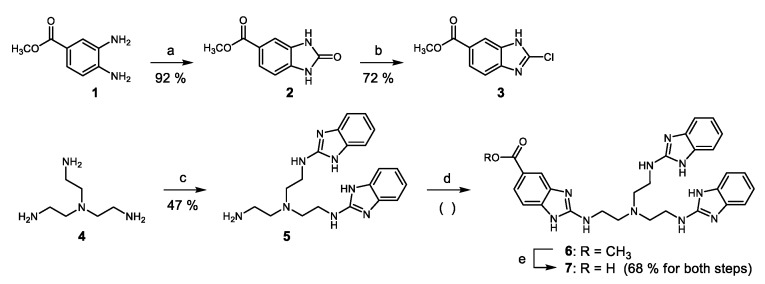
Synthesis of tris(2-aminobenzimidazole) **6**. Conditions: (**a**) urea, DMF, reflux, 3 h; (**b**) POCl_3_, reflux, 7 h; (**c**) 2-chloro benzimidazole, DIPEA, reflux, 4 h; (**d**) **3**, DIPEA, *n*BuOH, reflux, 8 h; (**e**) HCl, H_2_O, reflux, 6 h. DIPEA = diisopropylethylamine.

The initial method for the synthesis of tris(2-aminobenzimidazole) **6** suffered from the use of HgO to affect the formation of benzimidazoles from thiourea precursors [19]. Although HgO could be replaced by Mukaiyama’s reagent, the reduction and purification steps still remained tedious and time-consuming, resulting in limited product yields [13]. Our new method was based on the stepwise functionalization of tris(2-aminoethyl)amine (TREN, **4**) by nucleophilic displacement using 2-chlorobenzimidazole and building block **3** (Scheme 1). Although the yield of compound **5** was only 47%, the purification by chromatography was efficient. Partial Boc-protection of TREN, an essential step in the previous synthesis, was no longer required. Compound **3** was accessible in two simple steps from amino ester **1**. The reaction of **3** and **5** required *n*BuOH as a solvent. As a consequence, the crude reaction mixture of compound **6** (68%) contained some transesterification product (R = *n*Bu). However, in the final hydrolysis step, compound **6** and the butyl ester were both converted to carboxylic acid **7** in quantitative yield. Thus, we established a fast procedure for the synthesis of RNA cleaver **7** from TREN with 32% total yield and in batches of 13.5 g.

**Figure 1:**
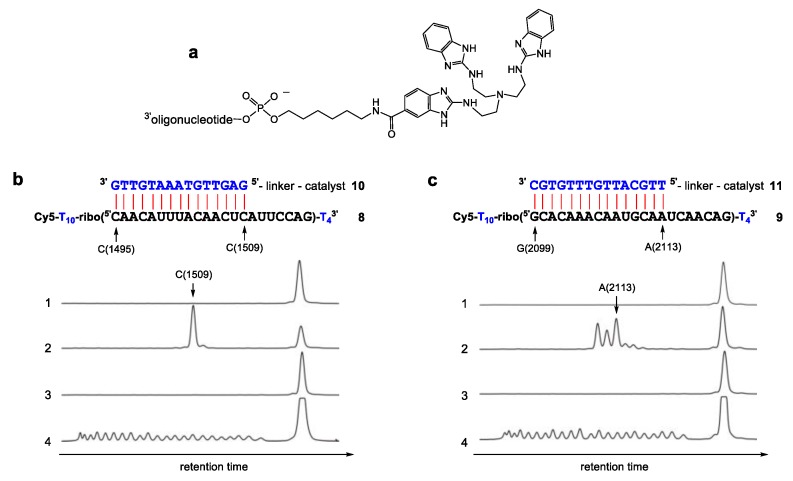
Cleavage of model RNAs. (**a**) Structure of the linker-catalyst part of conjugates **10** and **11**; (**b**) reaction of RNA **8** and conjugate **10**; (**c**) reaction of RNA **9** and conjugate **11**. Lanes 1: RNAs **8** or **9** before incubation. Lanes 2: RNAs **8** or **9** after incubation with complementary conjugates **10** and **11**, respectively (150 nM of RNA, 3 µM of conjugate, 50 mM Tris buffer (pH 8.0), 37 °C, 20 h). Lanes 3: Incubation of the noncomplementary pairs **8**/**11** or **9**/**10** under the same conditions. Lanes 4: Hydrolysis ladders of RNAs **8** and **9**. DNA and RNA nucleotides are shown in blue and black letters, respectively.

For the synthesis of the RNA cleaving conjugates **10** and **11**, the oligonucleotide strands were assembled on solid supports and modified at their 5′-ends with a C_6_ amino linker (Figure 1). After detritylation of the linker, carboxylic acid **7** was attached via amide formation, as described previously [12]. Both conjugates were removed from the support and deprotected by standard procedures. Yields of HPLC-purified conjugates were not higher than 30% in most cases, but the method worked reliably unless the amino linker became acylated by capping reagents during DNA strand assembly. This problem occurred with some commercial batches and led to complete failure of the conjugation step [20,21]. However, accidental capping of the amino linker could be unequivocally detected by mass spectrometry. The *PIM1* 3′-UTR regions of nt 1495–1509 and 2099–2113, harboring a validated siRNA site and a miR-33a target site, respectively, were chosen for our cleavage studies. Model substrates **8** and **9** were both RNA 22-mers extended by 10 (5′-end) and 4 (3′-end) thymidine residues and labeled with the fluorescent dye Cy5 at the 5′-end (Figure 1). The label allowed detection in an ALFexpress sequencer, while the thymidines helped to improve the resolution of electrophoretic bands. After chemical synthesis and purification, both oligonucleotides showed a clearly resolved single peak (Figure 1b,c, lanes 1). Hydrolysis ladders of **8** and **9** are depicted in lanes 4. Because only ribonucleotides are susceptible to base-induced cleavage, 22 fragments could be seen in each case.

Upon incubation with conjugate **10**, RNA **8** was cleaved with high selectivity at a single position (Figure 1b, Lanes 2). By coincidence with the hydrolysis ladder, the strand break could be located to the phosphodiester connecting C(1509) and A(1510), directly adjacent to the duplex formed by the conjugate and substrate. No reaction at all was observed when conjugate **10** was incubated with the noncomplementary RNA **9** (Figure 1c, Lanes 3). This demonstrated that hybridization was a prerequisite for cleavage and ruled out any cleavage by contaminating natural RNases. Reciprocally, conjugate **11** cleaved the complementary substrate **9** (Figure 1c, Lanes 2), but not RNA **8** (Figure 1b, Lanes 3). However, a more complex pattern was observed for the cleavage of substrate **9**. Apart from A(2113)-U at the end of the helix, cleavage was also seen within the duplex. Such effects are attributable to the fraying of A:T base pairs and were not observed when the closing base pair was G:C.

**Figure 2:**
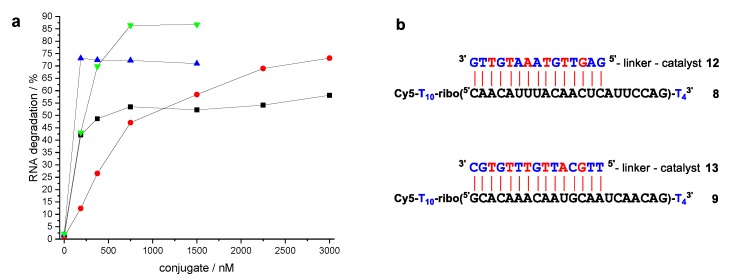
(**a**) Percentage of RNA cleavage after constant time as a function of conjugate concentration (150 nM of RNAs **8** or **9**, 50 mM Tris buffer (pH 8.0), 37 °C, 20 h). Red circles: RNA **8** and conjugate **10**. Green triangles: RNA **8** and DNA/LNA-mixmer conjugate **12**. Black squares: RNA **9** and DNA conjugate **11**. Blue triangles: RNA **9** and mixmer conjugate **13**. Data points are connected by lines for the sake of clarity. (**b**) Structures of mixmer conjugates **12** and **13**. DNA, LNA, and RNA nucleotides are shown in blue, red, and black letters, respectively.

**Figure 3:**
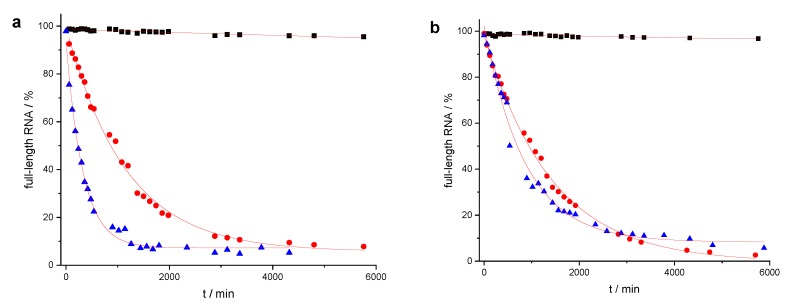
Determination of rate constants: 150 nM of RNAs **8** or **9** were incubated with 3 µM of **10** and **11**, or with 750 nM of **12** and **13** (50 mM Tris buffer (pH 8.0), 37 °C). The amount of residual full-length RNA is shown as a function of time. Representative examples of single experiments are shown, and the data were fitted to a first-order rate model (single exponential decay). Derived mean values of *k*_max_ (calculated from three independent experiments) were accurate within ±15%. (**a**) Reaction of RNA **8** with conjugates **10** (red circles) and **12** (blue triangles). (**b**) Reaction of RNA **9** with conjugates **11** (red circles) and **13** (blue triangles). Black squares: RNAs **8** and **9** incubated under the same conditions in the absence of conjugate.

For RNAs **8** and **9**, the amount of cleavage after incubation with increasing concentrations of conjugates **10** and **11** showed saturation, suggesting full occupation of the substrate strand (Figure 2). While 750 nM of **11** were sufficient to achieve maximum cleavage, distinctly higher amounts of **10** were required (3 µM). Thus, first-order rate constants were measured at concentrations of 3 µM for both conjugates (Figure 3). Substrate half-lives were 14 h (*k*_max_ = 0.050 h^−1^) for RNA **8** and 15 h (*k*_max_ = 0.046 h^−1^) for RNA **9**. In the next step, we replaced some of the DNA residues with locked nucleic acid (LNA) building blocks (DNA/LNA-mixmers) to increase substrate affinities (Figure 2). As expected, rate saturation now occurred at much lower concentrations of the corresponding conjugates **12** and **13,** while the cleavage patterns remained unchanged. The introduction of LNA building blocks also had a major impact on cleavage rates (150 nM of RNA, 750 nM of conjugate): The half-life of RNA **9** dropped to 10 h (*k*_max_ = 0.069 h^−1^). For RNA **8**, a half-life of only 3.5 h (*k*_max_ = 0.20 h^−1^) was observed. Tris(2-aminobenzimidazole) associates with tetrahedral oxyanions such as sulfate and phosphate. Therefore, the multitude of phosphorylated species in a cell might cause competitive inhibition of RNA cleavage. To exclude such effects, the reaction of **8** and **12** was also conducted in 50 mM of phosphate buffer at pH 8.0. The reaction rate, however, remained unchanged (data not shown).

The cleavage kinetics of transcripts was analyzed with three RNA substrates of increasing complexity: A 155-mer (**14**), as well as related 412- (**15**) and 430-mers (**17**), all three encompassing the siRNA 1491 site in the *PIM1* 3′-UTR (for details, see “Supplementary Materials”). In contrast to short oligoribonucleotide substrates, the target sites in such larger RNAs are embedded in complex overall structures that, in addition, form ensembles of near-isoenergetic and possibly alternative structures. This may affect the accessibility of the target site, the association kinetics of the antisense oligonucleotides carrying the chemical nuclease, and the extent of substrate cleavage: Also, larger RNA substrates may give rise to nonspecific interactions and cleavage events. The target region was predicted [22] to adopt a very similar fold in the 412/430-meric RNAs (**15**, **17**) and the full-length 3′-UTR, while it was predicted to be somewhat more structured in the 155-mer RNA **14** (Figures S16 and S17).

When RNA **15** was incubated with a large excess of conjugate **12** (250 nM **15**, 1 µM **12**, 50 mM Tris buffer (pH 8.0), 37 °C), fast cleavage occurred and two defined fragments appeared (Figure S15). Full occupation of the binding site and thus first-order kinetics could be expected under such conditions. A substrate half-life of roughly 2.5–3 h was measured that corresponded well with the cleavage kinetics observed for **12** and model RNA **8**. As in the model reaction, the addition of phosphate buffer (50 mM) had no impact on cleavage kinetics (Figure S15).

**Figure 4:**
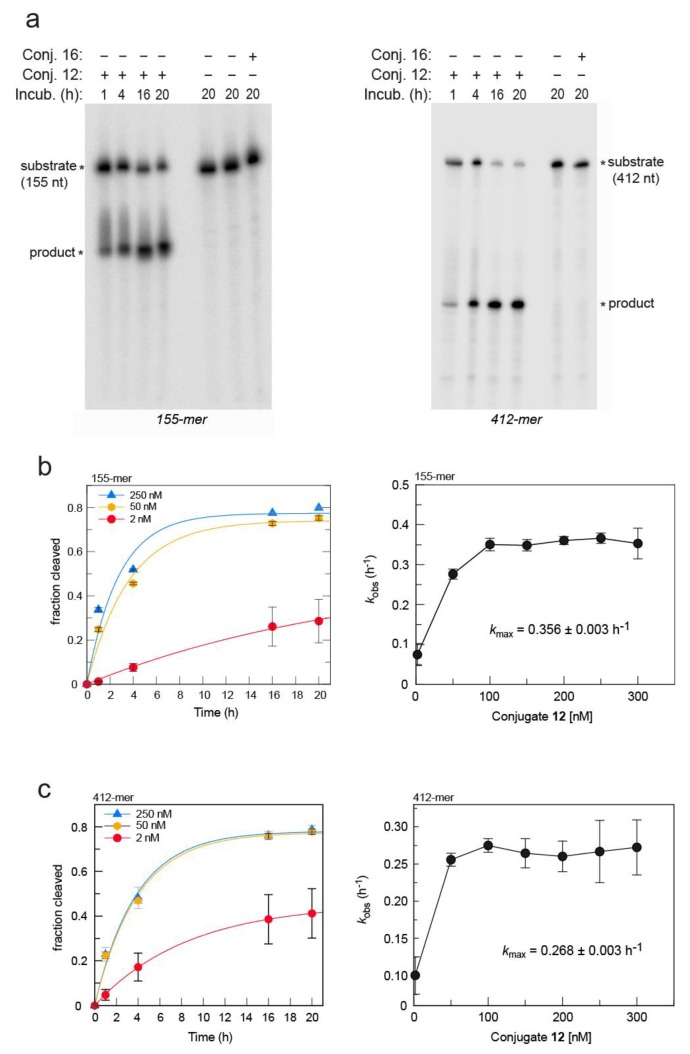
(**a**) Example gel images showing the time courses of cleavage of the RNA 155-mer **14** (left) and 412-mer **15** (right) by conjugate **12** at 37 °C. The 5′-^32^P-labeled 155-mer **14** and 412-mer **15** (~24 nM) were incubated with 300 nM (155-mer) and 200 nM (412-mer) of conjugate **12** for the indicated time periods. As a control, the RNA was incubated without conjugate or with the noncomplementary control conjugate **16** (cleaver-linker-5′-ctCcTgAcAaGgTat-3′; small letters: DNA; capital letters: LNA) for 20 h. (**b**) Examples of time courses (left image) of cleavage of the 155-mer **14** at three different concentrations of conjugate **12**, based on experiments, as shown in panel (**a**). Graph on the right: Plot of observed cleavage rate constant (*k*_obs_) as a function of conjugate **12** concentration (error bars: Standard deviation of the mean). Data points were connected by lines (no curve fitting), and the mean value of the *k*_obs_ values obtained at 100 to 300 nM of conjugate **12** were used to derive *k*_max_. (**c**) As in (**b**), but using the 412-mer substrate RNA **15**.

For a closer examination, the 155-mer **14** and 412-mer **15** were 5′ end-labeled with ^32^P. Incubation with conjugate **12** resulted in distinct cleavage products that were not observed in the absence of **12** or in the presence of the noncomplementary conjugate **16** (Figure 4a and Figures S9–S11). In the applied kinetic setup, the maximum cleavage rate constant (*k*_max_) was 0.36 h^−1^ for the 155-mer and 0.27 h^−1^ for the 412-mer (Figure 4b,c). These values were in the same range as those obtained for cleavage of the fluorescently labeled RNA **8** by conjugate **12** (0.2 h^−1^).

**Figure 5:**
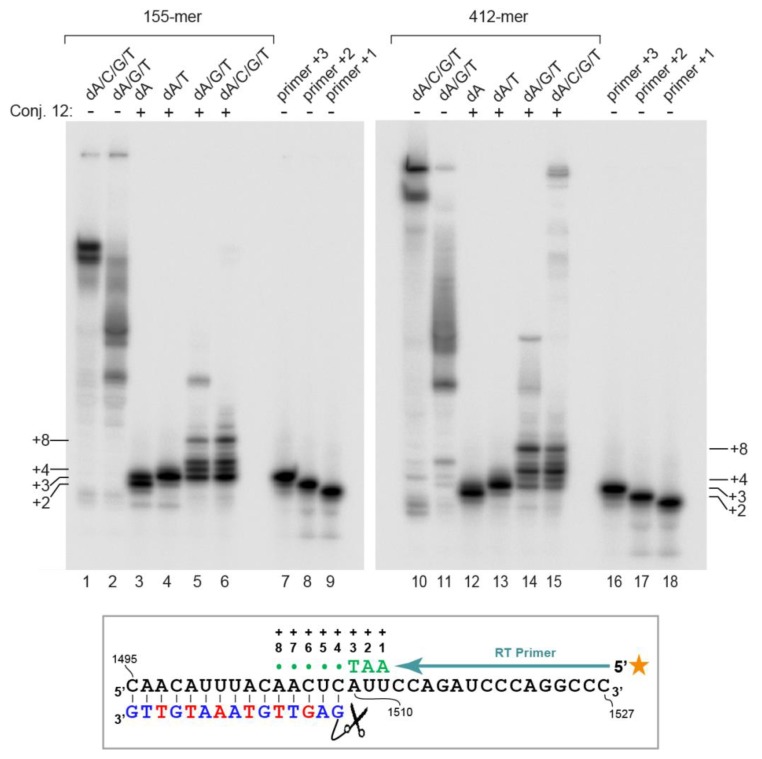
Primer extension assays to map the sites of cleavage on the RNA 155-mer **14** and 412-mer **15** catalyzed by conjugate **12**. Here, dA/C/G/T: Reverse transcription (RT) in the presence of 0.5 mM of each dNTP; dA/G/T: RT without dCTP; dA/T: RT without dCTP and dGTP; dA: RT in the presence of 0.5 mM dATP only. Lanes primer +1, +2, and +3: 5′-^32^P-end-labeled DNA oligonucleotides mimicking the +1, +2, and +3 extension products of the reverse transcription reaction (5′-GGGCCTGGGATCTGG**A**-3′, 5′-GGGCCTGGGATCTGG**AA**-3′, and 5′-GGGCCTGGGATCTGG**AAT**-3′). Note that in Lanes 3 (only dATP present), we saw not only the expected +2 extension product, but also a +3 product, suggesting that the reverse transcriptase added an additional untemplated nucleotide. The scheme at the bottom illustrates the target RNA region (black letters), the complementary conjugate **12** (blue: DNA residues, red: LNA residues), the chemical nuclease as scissors, the RT primer as a cyan arrow (the 5′-32P label indicated by the orange asterisk), and the first three primer extension products as green letters and the longer ones as green dots.

We further analyzed cleavage site selection on the 155- and 412-mer by primer extension. Here we used the uncleaved RNAs as controls (Figure 5, lanes 1, 2, 10, and 11). Note that a fraction of uncleaved substrate remained after 20 h of incubation with conjugate **12** (see Figure 4a). Thus, primer extension on the RNA substrates subjected to cleavage resulted in extension products derived from priming on cleaved substrate molecules (major fraction), but also yielded extension products derived from priming on uncleaved substrate molecules (minor fraction). A comparison of extension products in lanes corresponding to conjugate-treated versus untreated RNAs in the presence of all four dNTPs (Figure 5, cf. lanes 1 and 6, and lanes 10 and 15) clearly showed that reverse transcriptases acting on RNA molecules preincubated with conjugate **12** mainly terminated polymerization after the addition of 3, 4, 5, and 8 nucleotides. In contrast, the bulk of primers was extended to much longer products in the presence of untreated 155- and 412-mer. We conclude that the chemical nuclease, linker-coupled to the 5′-end of the antisense DNA/LNA mixmer, cleaved immediately adjacent to the duplex end involving the 5′-terminal nucleotide of the antisense mixmer (extension product +3; see Figure 5, bottom scheme, and Figure S16a,b), but also at internal sites of the antisense mixmer/target RNA duplex (the major cuts corresponding to extension products +4, +5, and +8).

**Figure 6:**
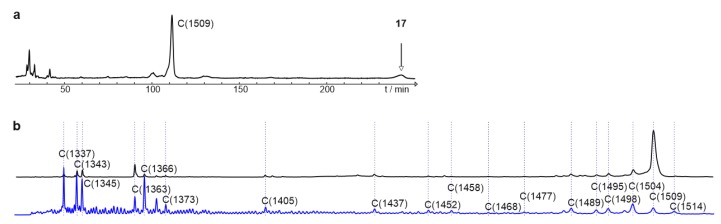
Cleavage of dye-labeled 430-mer **17** (250 nM) by conjugate **12** (1 µM), analyzed with an ALFexpress sequencer (50 mM Tris buffer (pH 8.0), 37 °C). (**a**) Overview: The peak of 430-mer **17** appeared at 242 min, a main cleavage fragment at 111 min (8% denaturing PAGE). (**b**) Analysis of the first 200 nt at higher resolution. Black line: RNA **17** after incubation with conjugate **12**. Blue line: Base ladder of RNA **17**. The minor peaks correspond to CA dinucleotide sites, known to be highly susceptible to hydrolysis. In the present experiment, this effect was helpful for the assignment of peaks (10% denaturing PAGE). Dotted lines were included to mark corresponding peaks in both electropherograms.

For localizing the cleavage positions in the 412-mer **15**, the dye-labeled oligonucleotide Cy5-T_10_-CGCCGAUC was ligated to the 5′ end of the 412-mer using T4 RNA ligase 2. The predicted secondary structure of the resulting 430-mer **17** around C(1509) was identical to that of the 412-mer and closely resembled that of the full-length UTR (Figure S17). When incubated with conjugate **12**, a major new fragment appeared. By comparison with the base ladder and peak counting, the cleavage site could be assigned to the phosphodiester connecting nucleotides 1509 and 1510 in the *PIM1* mRNA mimic (Figure 6). A minor cleavage product at C(1504) (Figure 6b) corresponded to the +8 extension product in Figure 5.

## 3. Discussion

Over the last two decades, gene silencing by siRNAs [23] has become an important approach in functional genomics. Alternatively, the targeted destruction of RNAs can be induced by so-called gapmers, chimeric antisense oligonucleotides combining modified building blocks with a central part consisting of DNA residues [24]. Both methods are based on the recruitment of cellular enzymes by synthetic oligonucleotides. The promise to achieve comparable effects by conjugates of oligonucleotides with simple synthetic catalysts has stimulated the development of artificial nucleases [1,2,3,4,5,6,7,8,9,10,11,12,13,14,15]. Such compounds may also find some applications as tools for the chemical manipulation of RNA. The usefulness of artificial nucleases depends on several factors, such as ease of synthesis, specificity, reaction rates, and activity within cells. Here we disclosed a convenient route to the trisbenzimidazole catalyst. The resulting conjugates had the advantage of being independent from metal ions that could be harmful in cellular environments. RNA cleavage is highly specific with both simple and complex substrates. It also does not suffer from inhibition by phosphate ions. In terms of reaction rates, our present conjugates lagged behind the activities of natural enzymes such as RNase H. However, as the mean duration of the cell cycle of human tumor cells is in the range of two days [25], our conjugates may well contribute to the efficiency of target RNA inactivation in such cells. It was interesting to find that a change from DNA oligonucleotides to DNA/LNA mixmers substantially accelerated RNA cleavage, even when an excess of each type of conjugate was used to enforce complete hybridization of the substrate strands. Thus, reaction kinetics not only depended on the global duplex stability, but also on the local rigidity close to the catalyst. As such local stabilities depend on the number and position of LNA building blocks, the kinetic efficiency of cleaver conjugates may be further improved by optimizing the LNA modification pattern. The here-observed impact of modified nucleosides on cleavage kinetics offers a chance for developing improved systems capable of downregulating specific proteins in a cell.

## 4. Materials and Methods

### 4.1. In Vitro Transcription of the 155-mer and 412-mer RNA Substrates ***14*** and ***15***

The sequence encoding the 155-mer RNA substrate **14** (Figure S16) was amplified from the human *PIM1* gene [18] using the forward primer 5′-AGg aat tc*T AAT ACG ACT CAC TAT TA*G GGA TCC TGC TGT ATG ATA TGG TG-3′ (EcoRI site in small letters, T7 class II promoter sequence in italics) and the reverse primer 5′-AGC ggt acc TAG GAC CCC TGG AGA GTC-3′ (KpnI site in small letters, see Figure S12). The PCR product was cleaved with EcoRI and KpnI and cloned into pUC19 cut with the same restriction enzymes. In a second PCR reaction, the recombinant plasmid was amplified using two primers with a gap between their 5′-ends and which were elongated in an inverted direction (primer 1: 5′-GCG ATG CTT GAT ACA GGA ACA ACA T-3′; primer 2: 5′-TGC TCG AAA GGA ATA TCT CCA CAC AC-3′), as described before [26] (Figure S12). The linear copies of the plasmid were digested with DpnI (to destroy the original template DNA), 5′-phosphorylated with T4 polynucleotide kinase, and circularized with T4 DNA ligase for transformation of *E. coli* cells. The resulting recombinant plasmid was linearized with KpnI and utilized as a template for T7 in vitro transcription using in house-prepared enzyme essentially as described [27] (protocol 1 therein, but containing 10 mM dithiothreitol (DTT) and 60 µg/mL plasmid DNA). Transcription products were purified by phenol extraction using Roti^®^-Aqua-Phenol (Carl Roth, Karlsruhe, Germany), followed by chloroform extraction. The aqueous phase was then purified by 10% denaturing (8 M urea) polyacrylamide gel electrophoresis (PAGE), and the RNA band of interest was localized by UV shadowing and excised from the gel. Elution from gel slices was performed by shaking overnight at 4 °C in 1 M of NaOAc (pH 5.0). The eluted RNA was precipitated by ethanol for 1 h at −20 °C. After centrifugation (60 min at 13,800× *g* at 4 °C), the supernatant was discarded and the pellet was dissolved in ddH_2_O. The predicted structure of the RNA 155-mer is shown in Figure S16a.

The DNA template for 412-mer RNA **15** (cloned into pUC57, GenScript, Leiden, Netherlands) encoded a 304-nt fragment of the *PIM1* mRNA, including the UAG stop codon and the nearby siRNA 1491 site in the 3′-UTR. The second A residue of this fragment was mutated to G (for T7 transcription efficiency): A T7 promotor was added as well as EcoRV cleavage sites required for insertion into plasmid pUC57 (Figures S13 and S14). The final vector was then transformed into *E. coli*, amplified, and purified. From this plasmid, a PCR fragment was prepared encoding a 304-mer RNA using primers 5′-GTTTTCCCAGTCACGACGTT-3′ (forward) and 5′-GTCCAGCAATGCTGGACAC-3′ (reverse; both from Eurogentec, Liège, Belgium). In vitro transcription (see above) from this PCR product terminated prematurely and did not form the required RNA. However, when we extended the template by 108 nucleotides derived from the pUC57 plasmid using primers 5′-GTTTTCCCAGTCACGACGTT-3′ (forward) and 5′-TGTGGAATTGTGAGCGGATA-3′ (reverse; both from Biospring, Frankfurt, Germany), we could then efficiently transcribe the 412-mer RNA **15** from this new PCR fragment. The positioning of primers is shown in Figures S13 and S14. The 412-mer **15** (Figure S16b) was synthesized with the NEB HiScribeT7 High Yield RNA Synthesis Kit (New England Biolabs, Ipswich, MA, USA) using the PCR product described above. RNA purification was done as described above for the 155-mer, except that a 7% denaturing PAA gel was used. A GMP-primed analog of 412-mer **15**, used for the preparation of 430-mer **17**, was transcribed in vitro as follows: 25 ng/µL template, 7.5 mM of each NTP, 30 mM GMP, and 70 µg/mL T7 RNA polymerase (in house preparation) were incubated for 4 h at 37 °C in transcription buffer (80 mM HEPES-KOH, pH 7.5 at 25 °C, 24 mM MgCl_2_, 2 mM spermidine, 40 mM DTT). RNA purification was carried out using the Isolate II RNA mini kit (Bioline, Luckenwalde, Germany).

### 4.2. Dephosphorylation and 5′-[^32^P]-End-Labeling of RNA

RNAs **14** and **15** (~15 µg) were dephosphorylated for 25 min at 37 °C in 10 µL 1× FastAP buffer (10 mM Tris-HCl (pH 8 at 37 °C), 5 mM MgCl_2_, 100 mM KCl, 0.02% Triton X-100, 0.1 mg/mL BSA) containing 1 U of the thermosensitive alkaline phosphatase (FastAP, Thermo Fisher Scientific, Waltham, MA, USA). The enzyme was then inactivated by incubation for 5 min at 75 °C. For 5′-[^32^P]-end-labeling, 60 pmol of dephosphorylated RNA was incubated with 10 U T4 polynucleotide kinase (T4 PNK; Thermo Fisher Scientific) and 2 µL [γ^32^P]-ATP (3000 Ci/mmol, 10 µCi/µL) in 15 µL 1× reaction buffer A (50 mM Tris-HCl (pH 7.6 at 25 °C), 10 mM MgCl_2_, 5 mM DTT, 0.1 mM spermidine) for 60 min at 37 °C. The reaction was stopped by the addition of an equal volume of 2× RNA sample buffer (2× TBE buffer, 2.6 M urea, 66% deionized formamide, 0.02% (*w*/*v*) bromophenol blue, and 0.02% (*w*/*v*) xylene cyanol), and samples were purified by 8% denaturing (8 M urea) PAGE in the case of the 155-mer **14**. The labeled RNA was localized by autoradiography and eluted overnight in 1 M NaOAc (pH 5.0) at 8 °C. After filtration (centrifugal filter, 0.2 µm; VWR, Darmstadt, Germany), the RNA was precipitated with ethanol, redissolved in ddH_2_O, and stored at −20 °C. In the case of the 412-mer **15**, the gel purification step was omitted, as elution of the radiolabeled RNA from the gel was inefficient. Here, the transcript was passed over Illustra Microspin™ G-25 columns (GE Healthcare, Chicago, IL, USA) to remove low molecular weight components.

### 4.3. In Vitro Cleavage Assay

Cleavage studies with dye-labeled RNAs **8**, **9**, and **17** were executed as described previously [12]. For experiments shown in Figure 6, 8% and 10% denaturing PAGE gels were used. In addition, 5′-[^32^P]-labeled RNAs were refolded by preincubation in 50 mM Tris buffer pH 8.0 (3 min at 95 °C, 2 min at 85 °C, 2 min at 75 °C, 2 min at 65 °C, 2 min at 55 °C, and 3 min at 37 °C). Immediately after preincubation, the 5′-[^32^P]-RNA substrate (final concentration ~24 nM) and varying amounts of conjugate **12** (2 to 300 nM) were combined at 37 °C in a total volume of 20 µL 50 mM Tris buffer (pH 8.0) to initiate the cleavage reaction. Aliquots of 5 µL were withdrawn after 1, 4, 16, and 20 h of incubation at 37 °C and stopped by the addition of 5 µL 2× RNA sample buffer (see above). Samples without conjugate or containing the noncomplementary conjugate **16** (Figures S9–S11) served as controls. Cleavage products were then separated by 10% or 7% denaturing PAGE gel and visualized and quantified by phosphoimaging using a Bio-Imaging Analyzer FLA3000-2R (Fujifilm, Minato, Japan).

### 4.4. Primer Extension

Primer extension analysis was performed with 1–2 µg of the respective T7 in vitro transcripts (155-mer or 412-mer). Beforehand, RNAs were incubated for 20 h either in the presence or absence of 12.5 µM conjugate **12**. The 5′-^32^P-end-labeled primer (5′-GGGCCTGGGATCTGG-3′) was annealed to the uncleaved and cleaved RNA transcripts according to the SuperScript^TM^ III Reverse Transciptase protocol supplied by the manufacturer (Thermo Fisher Scientific). Reverse transcription was then performed for 50 min at 55 °C following the protocol of the manufacturer. Reactions were stopped by the addition of 20 µL 2× RNA sample buffer (see above) and separated on a 20% denaturing (8 M urea) PAA gel.

### 4.5. Splint Ligation Producing RNA **17**

A GMP-primed analog of 412-mer **15**, the dye-labeled oligonucleotide 5′-Cy5-TTTTTTTTTTCGCCGAUC-3′, and the DNA splint oligonucleotide 5′-GAGGTGGACCGATCGGCG-3′ (both from Eurogentec) were mixed at a ratio 1:1:1.5 in ligase buffer (50 mM Tris-HCl buffer (pH 7.5) containing 2 mM MgCl_2_, 1 mM DTT, and 400 µM ATP). Poly(vinylsulfonic acid) (Sigma-Aldrich, St. Louis, MO, USA), an inhibitor for RNase A [28], was added at a concentration of 5 mg/mL to the ligation reaction. The annealing, ligation reaction, and purification of RNA were carried out as described [29].

## Supplementary Materials

Synthesis of catalyst **7**, of oligonucleotide conjugates **10**–**13** and **16**. PCR strategies. Cleavage assay with unlabeled RNA **15**. Predicted secondary structures of *PIM1* model transcripts. NMR spectra.
