# Supplementary material for: Site-Specific Cleavage of RNAs Derived from the PIM1 3′-UTR by a Metal-Free Artificial Ribonuclease
Source: Molecules. 2019 Feb 23;24(4):807. doi: 10.3390/molecules24040807 (PMC6412833; doi:10.3390/molecules24040807)
Supplement: Supplementary file 1 [file molecules-24-00807-s001.pdf]

Site-Specific Cleavage of RNAs Derived from the *PIM1* 3′-UTR by a Metal-Free Artificial Ribonuclease

Felix Zellmann,[a] Laura Thomas,[b] Ute Scheffer,[a] Roland K. Hartmann,[b] Michael W. Göbel*[a]

[a] Institute of Organic Chemistry and Chemical Biology, Goethe University Frankfurt
Max-von-Laue-Str. 7, D-60438 Frankfurt am Main, Germany
[b] Institute of Pharmaceutical Chemistry, Philipps University Marburg,
Marbacher Weg 6-10, D-35032 Marburg, Germany
Email: Michael W. Göbel – m.goebel@chemie.uni-frankfurt.de
* Corresponding author

Supplementary material

Table of contents:

General S1
Synthesis of the tris(2-aminobenzimidazole) derivative **7** S2
Synthesis, purification, quantification, and analysis of conjugates S5
Mass spectra and HPLC plots of conjugates S6
Preparation of RNA transcripts **14** and **15** S11
Cleavage assay with unlabeled 412-mer **15** S13
Secondary structure predictions S14
References S18
NMR spectra S19

General

All chemicals were reagent grade and used as purchased. All of the reactions except ester hydrolysis were performed under an argon atmosphere. Reactions were monitored by TLC using Merck TLC silica gel 60 F-254 aluminum sheets. Compounds were visualized by UV light (254 and 366 nm) or by staining with a solution of ninhydrin in ethanol. Column chromatography was carried out on silica gel 60 (0.04 - 0.063 mm). Proton nuclear magnetic resonance (^{1}H-NMR) spectra and carbon nuclear magnetic resonance (^{13}C-NMR) were recorded at 300 K with *Bruker* AV 300 (^{1}H: 300 MHz; ^{13}C: 75.5 MHz) and *Bruker* AV 500 (^{1}H: 500 MHz; ^{13}C: 125.8 MHz) NMR spectrometers. Chemical shifts for protons are reported in parts per million (δ scale) and internally referenced to the proton resonances of the solvent

(DMSO-d_6: δ 2.50). Chemical shifts for carbon are reported in parts per million (δ scale) and referenced to the carbon resonances of the solvent (DMSO-d_6: δ 39.52). Data are represented as follows: chemical shift, multiplicity (s = singlet, bs = broad singlet, d = doublet, bd = broad doublet, t = triplet, q = quartet, m = multiplet, dd = double doublet), coupling constants in Hz, and integration. IR spectra were recorded with a Jasco FT/IR-420 spectrometer equipped with an ATR unit. Melting points (uncorrected) were determined on a Schorpp apparatus MPM-H2. ESI-MS spectra were obtained on a Fisons VG Plattform II. HRMS spectra were recorded on a MALDI LTQ Orbitrap mass spectrometer from Thermo Scientific.

Synthesis of the tris(2-aminobenzimidazole) derivative 7.

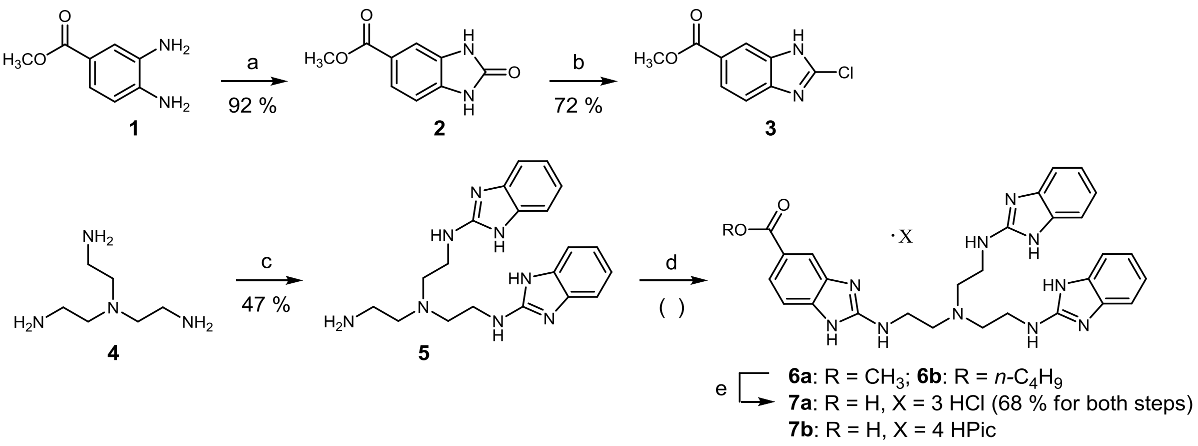

Scheme 1. Synthesis of tris(2-aminobenzimidazole) **7**. Conditions: (**a**) urea, DMF, reflux, 3 h; (**b**) $POCl_3$, reflux, 7 h; (**c**) 2-chloro benzimidazole, DIPEA, reflux, 4 h; (**d**) **3**, DIPEA, *n*BuOH, reflux, 8 h; (**e**) HCl, H_2O, reflux, 6 h.

Methyl 2-oxo-2,3-dihydro-1*H*-benzo[*d*]imidazole-5-carboxylate (2).

A suspension of methyl 3,4-diaminobenzoate **1** (25.0 g, 0.151 mol, 1 eq) and urea (27.2 g, 0.453 mol, 3 eq) in DMF (200 mL) was heated to reflux for 3 h. The solvent was removed under reduced pressure and the residue was suspended in water (200 mL). The solid was collected by filtration, repeatedly washed with water and dried *in vacuo* to obtain a light brown solid. The crude product obtained this way was of high purity and could be used in the next step without further purification (26.7 g, 0.139 mol, 92 %). R_f = 0.37 (DCM/MeOH 20:1). ^{1}H NMR (300 MHz, DMSO-d_6): δ [ppm] = 10.91 (bs, 2 H, N*H*), 7.63 (dd, *J* = 8.2 Hz, *J* = 1.6 Hz, 1 H, 6-*H*), 7.47 (d, *J* = 1.6 Hz, 1 H, 4-*H*), 7.02 (d, *J* = 8.2 Hz, 1 H, 7-*H*), 3.81 (s, 3 H, C*H*$_3$). ^{13}C NMR (75.5 MHz, DMSO-d_6): δ [ppm] = 166.4, 155.4, 134.0, 129.6, 122.9, 121.8, 109.0, 108.2, 51.9. IR: $\tilde{\nu}$ [cm^{-1}] = 2983 (w), 1759 (m), 1714 (s), 1433 (m), 1300 (s), 1272 (s), 1226 (s), 1113 (m), 1082 (s), 1028 (m), 964 (m), 929 (w), 877 (m), 753 (s), 714 (s), 681 (s), 604 (s), 552 (m), 478 (m).

MS (ESI-): m/z (%) = 191.07 (100) [M-H]$^+$, calcd for $C_9H_7N_2O_3$: 191.05. HRMS (MALDI): m/z = 193.06065 [M+H]$^+$, calcd for $C_9H_8N_2O_3$+H^+: 193.06077.

Methyl 2-chloro-1*H*-benzo[*d*]imidazole-5-carboxylate (3).

A suspension of benzimidazolone **2** (25.0 g, 0.130 mol, 1 eq) in $POCl_3$ (150 mL) was heated to reflux for 7 h. The excess of $POCl_3$ was evaporated and the residue was neutralized with saturated $NaHCO_3$ solution. The precipitate was collected by filtration and dried *in vacuo*. The aqueous phase was extracted with DCM, dried over $MgSO_4$ and the solvent was removed under reduced pressure. The light brown solids obtained this way were of high purity and could be used in the next step without further purification (19.7 g, 93.6 mmol, 72 %). R_f = 0.78 (EtOAc). Mp: 174 - 175 °C. ^{1}H NMR (300 MHz, DMSO-*d*$_6$): δ [ppm] = 8.1 (bs, 1 H, 4-*H*), 7.86 (dd, *J* = 8.5 Hz, *J* = 1.6 Hz, 1 H, 6-*H*), 7.60 (bd, *J* = 8.5 Hz, 1 H, 7-*H*), 3.86 (s, 3 H, C*H*$_3$). ^{13}C NMR (75.5 MHz, DMSO-*d*$_6$): δ [ppm] = 166.4, 141.3, 123.8, 123.6, 52.1. IR: $\tilde{\nu}$ [cm^{-1}] = 2638 (w), 1717 (s), 1627 (w), 1432 (s), 1362 (w), 1296 (s), 1284 (s), 1231 (s), 1207 (s), 1124 (w), 1086 (m), 987 (m), 891 (w), 828 (w), 805 (w), 765 (s), 742 (s), 621 (w), 535 (w), 473 (w). HRMS (MALDI): m/z = 211.02687 [M+H]$^+$, calcd for $C_9H_7ClN_2O_2$+H^+: 211.02688.

***N*^{1}-(2-((1*H*-Benzo[*d*]imidazol-2-yl)amino)ethyl)-*N*^{1}-(2-aminoethyl)-*N*^{2}-(1*H*-benzo[*d*]-imidazol-2-yl)ethane-1,2-diamine (5).**

A suspension of TREN **4** (10.0 g, 68.4 mmol, 1 eq), 2-chloro benzimidazole (15.7 g, 0.103 mol, 1.5 eq) and DIPEA (17.9 mL, 13.3 g, 0.103 mol, 1.5 eq) was heated to reflux for 4 h. The solvent was removed under reduced pressure, the solid residue was adsorbed to 150 g of silica and purified via column chromatography (DCM/MeOH 10:1, 2 % $NH_{3(aq)}$) → DCM/MeOH 2:1, 2 % $NH_{3(aq)}$) to obtain the product as a yellow foam (12.1 g, 32.0 mmol, 47 %). R_f = 0.39 (DCM/MeOH 5:1, 2 % $NH_{3(aq)}$). ^{1}H NMR (300 MHz, DMSO-*d*$_6$): δ [ppm] = 7.15 - 7.08 (m, 4 H), 6.88 - 6.81 (m, 4 H), 3.36 (t, *J* = 6.2 Hz, 4 H), 2.77 - 2.62 (m, 8 H). ^{13}C NMR (75.5 MHz, DMSO-*d*$_6$): δ [ppm] = 155.7, 138.6, 119.0, 111.5, 54.3, 53.9, 40.5, 38.4. MS (ESI-): m/z (%) = 377.22 (100) [M-H]$^-$, calcd for $C_{20}H_{25}N_8$: 377.22. HRMS (MALDI): m/z = 379.23527 [M+H]$^+$, calcd for $C_{20}H_{26}N_8$+H^+: 379.23532.

Methyl- and *n*-butyl 2-((2-(bis(2-((1*H*-benzo[*d*]imidazol-2-yl)amino)ethyl)amino)ethyl)-amino)-1*H*-benzo[*d*]imidazole-5-carboxylate (6a, 6b).

A suspension of disubstituted TREN **5** (11.6 g, 30.6 mmol, 1 eq), 2-chloro benzimidazole methyl carboxylate **3** (7.10 g, 33.7 mmol, 1.1 eq) and DIPEA (5.9 mL, 4.35 g, 33.7 mmol, 1.1 eq) in *n*BuOH (40 mL) was heated to reflux for 8 h. The solvent was evaporated under reduced pressure and the residue was purified via column chromatography (DCM/MeOH

15:1, 2 % $NH_{3(aq)}$ → DCM/MeOH 10:1, 2 % $NH_{3(aq)}$) to obtain a beige solid (11.5 g mixture of methyl and butyl carboxylates + 2.22 g (5.87 mmol) of reisolated compound **5**).

By column chromatography, some fractions of pure **6a** could be obtained:

Methyl carboxylate **6a**: R_f = 0.29 (DCM/MeOH 10:1, 2 % $NH_{3(aq)}$). ^{1}H NMR (300 MHz, DMSO-d_6): δ [ppm] = 7.71 (dd, *J* = 1.7 Hz, *J* = 0.5 Hz, 1 H, 4-*H*), 7.58 (dd, *J* = 8.3 Hz, *J* = 1.7 Hz, 1 H, 6-*H*), 7.16 (dd, *J* = 8.3 Hz, *J* = 0.5 Hz, 1 H, 7-*H*), 7.14 - 7.08 (m, 4 H), 6.94 (bs, 1 H, N*H*), 6.88 - 6.82 (m, 4 H), 6.54 (bs, 2 H, N*H*), 3.79 (s, 3 H, C*H*$_3$), 3.45 - 3.37 (m, 6 H), 2.78 (t, *J* = 6.7 Hz, 6 H). MS (ESI+): m/z (%) = 553.14 (100) $[M+H]^+$, calcd for $C_{29}H_{33}N_{10}O_2$: 553.28. HRMS (MALDI): m/z = 553.28078 $[M+H]^+$, calcd for $C_{29}H_{32}N_{10}O_2+H^+$: 553.27825.

In the NMR spectra of mixtures of **6a** and **6b** signals could be assigned to ester **6b**:

Butyl carboxylate **6b**: R_f = 0.34 (DCM/MeOH 10:1, 2 % $NH_{3(aq)}$). ^{1}H NMR (300 MHz, DMSO-d_6): δ [ppm] = 7.71 (dd, *J* = 1.7 Hz, *J* = 0.5 Hz, 1 H, 4-*H*), 7.58 (dd, *J* = 8.3 Hz, *J* = 1.7 Hz, 1 H, 6-*H*), 7.16 (dd, *J* = 8.3 Hz, *J* = 0.5 Hz, 1 H, 7-*H*), 7.14 - 7.08 (m, 4 H), 6.94 (bs, 1 H, N*H*), 6.88 - 6.82 (m, 4H), 6.54 (bs, 2 H, N*H*), 4.21 (t, *J* = 6.4 Hz, 2 H, COOC*H*$_2$), 3.45 - 3.37 (m, 6 H), 2.78 (t, *J* = 6.7 Hz, 6 H), 1.71 - 1.62 (m, 2 H), 1.49- 1.34 (m, 2 H), 0.93 (t, *J* = 7.3 Hz, 3 H, C*H*$_3$). MS (ESI+): m/z (%) = 595.20 (100) $[M+H]^+$, calcd for $C_{32}H_{39}N_{10}O_2$: 595.33. HRMS (MALDI): m/z = 595.32793 $[M+H]^+$, calcd for $C_{32}H_{38}N_{10}O_2+H^+$: 595.32520.

^{13}C NMR (75.5 MHz, DMSO-d_6, mixture of two carboxylates): δ [ppm] = 167.1, 157.6, 155.6, 121.7, 119.9, 119.1, 111.7, 111.4, 53.7, 53.6, 51.5, 40.6. IR (mixture of two carboxylates): $\tilde{\nu}$ [cm^{-1}] = 2971 (m), 2904 (m), 2755 (m), 2656 (m), 1658 (s), 1619 (s), 1476 (m), 1436 (m), 1389 (m), 1289 (m), 1232 (m), 1205 (m), 1139 (m), 1087 (m), 1015 (w), 982 (w), 897 (w), 738 (m), 685 (m), 619 (m).

Hydrochloride of 2-((2-(bis(2-((1*H*-benzo[*d*]imidazol-2-yl)amino)ethyl)amino)ethyl)-amino)-1*H*-benzo[*d*]imidazole-5-carboxylic acid (7a).

A suspension of esters **6a/b** (11.5 g) in 6 M hydrochloric acid (150 mL) was heated to reflux for 6 h. The solvent was removed under reduced pressure and the residue was dried *in vacuo* to obtain a light brown solid. The crude product could be used in the conjugation experiments without further purification (13.5 g of trishydrochloride, 20.8 mmol, 68 % for two steps). For analytical purposes a small amount was dissolved in MeOH and treated with a saturated solution of picric acid in MeOH until no more precipitate formed. The solid was collected by filtration, washed thoroughly with MeOH and dried *in vacuo*. The picrate salt was converted into the corresponding hydro chloride using DOWEX ion exchange resin (Cl^--form) with DMSO/MeOH as an eluent.

Hydrochloride **7a**: ^{1}H NMR (500 MHz, DMSO-d_6): δ [ppm] = 13.23 (very broad singlet, N*H*), 9.49 (bs, 1 H, CH_2N*H*), 9.21 (bs, 2 H, CH_2N*H*), 7.90 (d, 1.5 Hz, 1 H, 4-*H*), 7.82 (dd, *J* = 8.3 Hz, *J* = 1.6 Hz, 1 H, 6-*H*), 7.44 (d, *J* = 8.3 Hz, 1 H, 7-*H*), 7.39 - 7.35 (m, 4 H), 7.21 - 7.18 (m, 4 H), 3.83 (bs, 6 H), 3.23 (bs, 6 H). ^{13}C NMR (125.8 MHz, DMSO-d_6): δ [ppm] = 167.0, 151.0, 150.0, 133.5, 130.0, 129.7, 125.5, 124.8, 123.0, 112.3, 111.3, 111.0, 52.1, 42.1. IR: $\tilde{\nu}$ [cm^{-1}] = 2898 (m), 1658 (s), 1619 (m), 1474 (m), 1386 (m), 1264 (m), 1197 (m), 1013 (w), 897 (w), 841 (w), 739 (m), 690 (w). MS (ESI+): m/z (%) = 539.14 (100) [M+H]$^+$, calcd for $C_{28}H_{31}N_{10}O_2$: 539.26. HRMS (MALDI): m/z = 539.26041 [M+H]$^+$, calcd for $C_{16}H_{34}N_4O_4$+H^+: 539.26260.

Picrate **7b**: ^{1}H NMR (500 MHz, DMSO-d_6): δ [ppm] = 12.61 (very broad singlet, N*H*), 8.94 (bs, 1 H, CH_2N*H*), 8.73 (bs, 2 H, CH_2N*H*), 8.56 (s, 8 H, *H*Pic), 7.84 - 7.82 (m, 2 H, 4-*H*, 6-*H*), 7.39 (d, *J* = 8.8 Hz, 1 H, 7-*H*), 7.35 - 7.31 (m, 4 H), 7.23 - 7.20 (m, 4 H), 3.58 (bs, 6 H), 3.06 (bs, 6 H). ^{13}C NMR (125.8 MHz, DMSO-d_6): δ [ppm] = 166.9, 160.9, 151.3, 150.1, 141.7, 133.4, 129.9, 129.6, 125.8, 125.3, 125.0, 124.5, 123.3, 112.2, 111.3, 111.1, 51.6.

Synthesis, purification, quantification, and analysis of conjugates.

General: Reversed Phase (RP) HPLC was executed on a Jasco LC-900 HPLC system equipped with a Jasco UV-975 detector (detection at 254 nm) and a semi preparative column Phenomenex Gemini C18 10 µm 110 Å (250 x 10 mm). All conjugates were concentrated in a SpeedVac (Christ or Savant). Water was treated with DEPC and autoclaved.

Synthesis of DNA or mixmer conjugates and cleavage studies: For experimental details see [1]. Typical conjugation yields are between 20 and 30 % and may be further limited by accidental acylation of the amino linker due to capping reagents and/or acyl migration.

Isolation and purification: For experimental details see [1]. RP-HPLC: A: 1 M TEAA buffer pH 7.0, B: acetonitrile, C: DEPC-H_2O. Gradient: constant 10 % A, 1 % B from 0-1min, 1-30 % from 1-30 min; flow 4 mL/min; column temperature 50 °C.

Quantification: Oligonucleotide concentrations were determined via UV spectrometry on a nanodrop2000 (Thermo Scientific) using Lambert-Beer´s law. Extinction coefficients were calculated by a nearest neighbor model according to literature [2]. For simplification, influences of tris(2-aminobenzimidazole) were neglected.

Mass spectrometry: Oligonucleotides were analyzed via ESI mass spectrometry using a LCMS instrument with microTOF-Q II analyser (Bruker). An Agilent 1200 Series HPLC using methanol/0.005 M TEAA buffer (gradient 0-60 %) was applied as LC system.

Mass spectra and HPLC plots of conjugates.

Conjugate **10** calculated exact mass: 5361.1; found 5363.7; 5382.7 (+ Na^{+}).

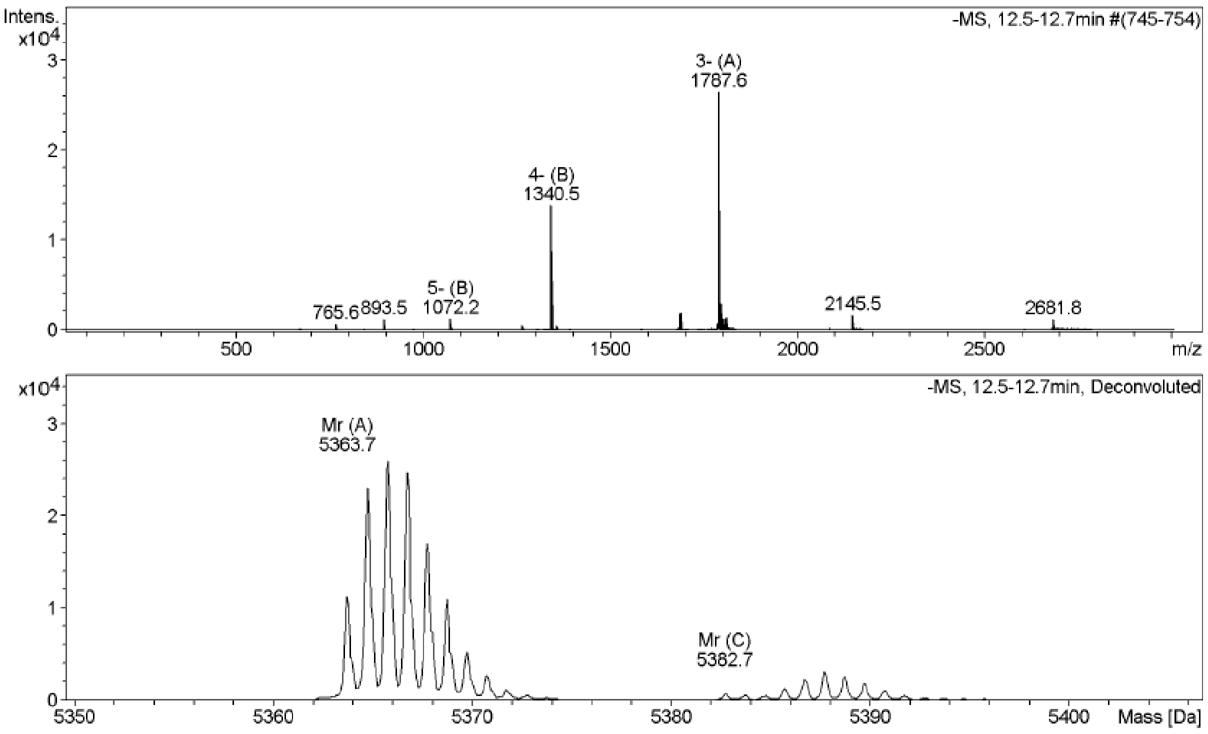

Figure S1: LC-MS analysis of conjugate **10**.

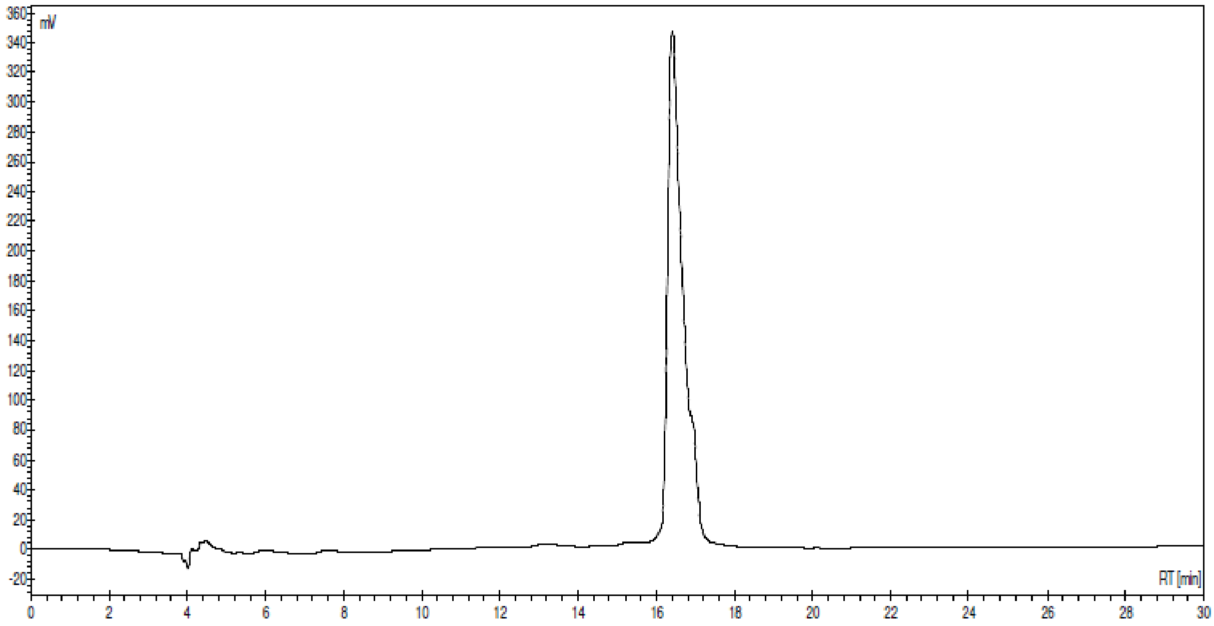

Figure S2: Chromatogram of conjugate **10** after purification.

Conjugate **11** calculated exact mass: 5279.7; found 5281.6; 5300.6 (+ Na^{+}).

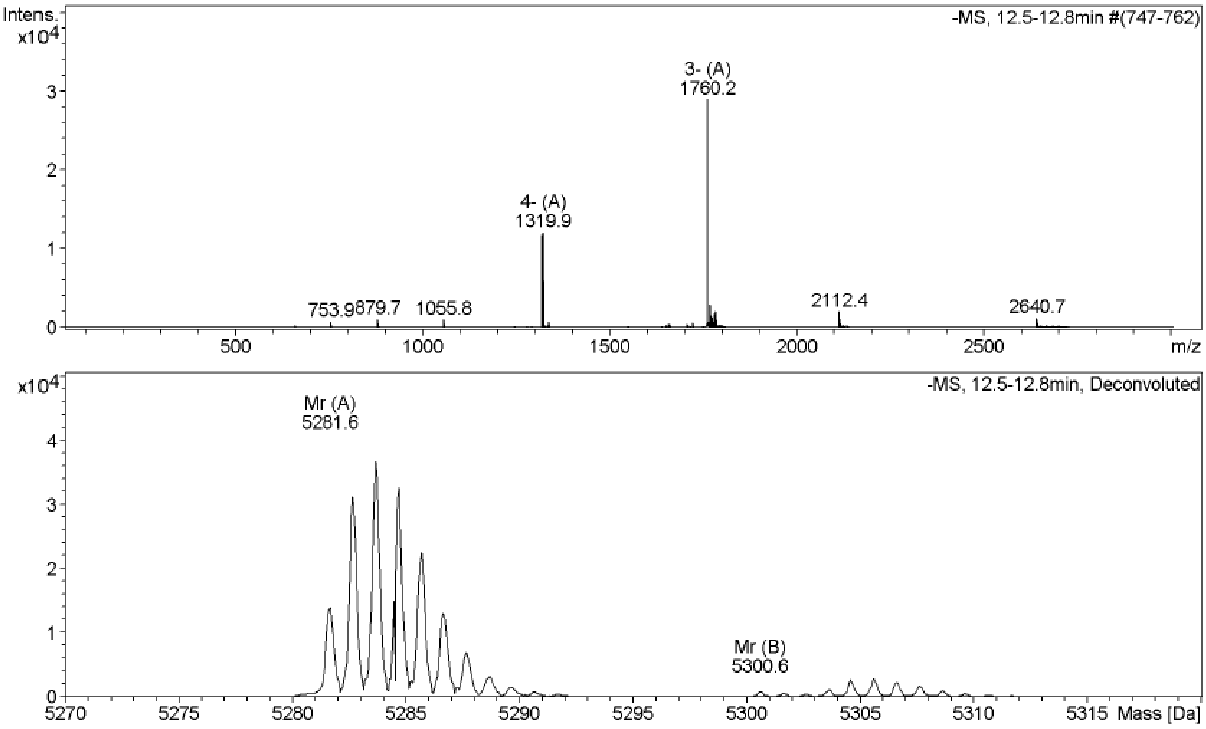

Figure S3: LC-MS analysis of conjugate **11**.

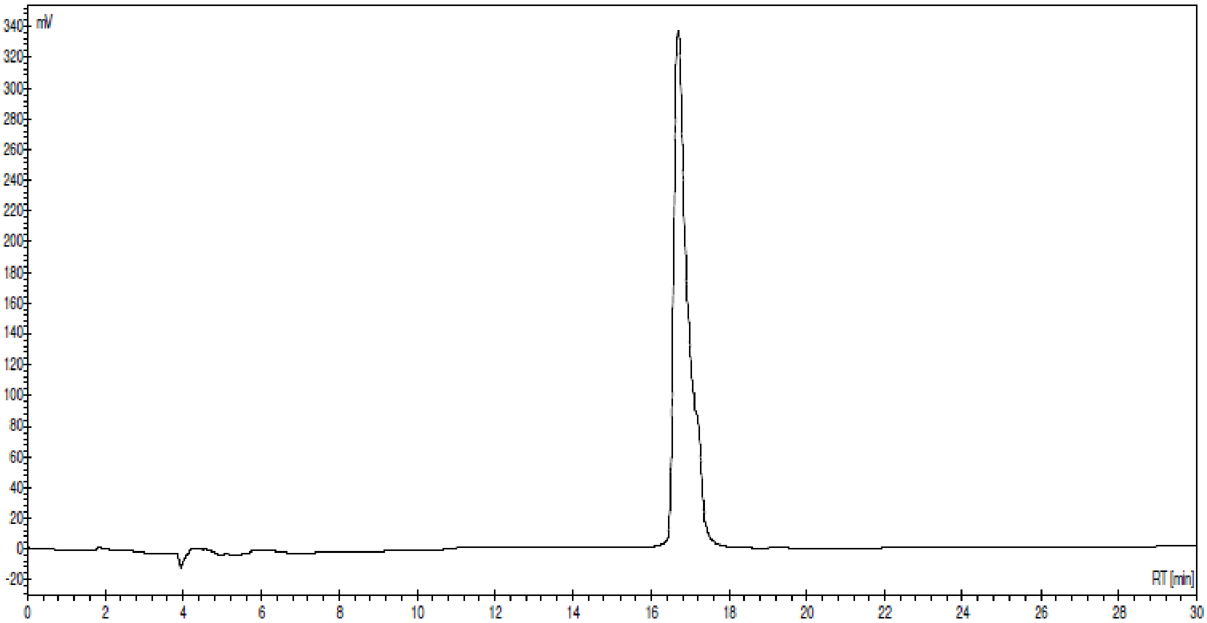

Figure S4: Chromatogram of conjugate **11** after purification.

Conjugate **12** calculated exact mass: 5529.1; found 5526.9; 5549.8 (+ Na^{+}).

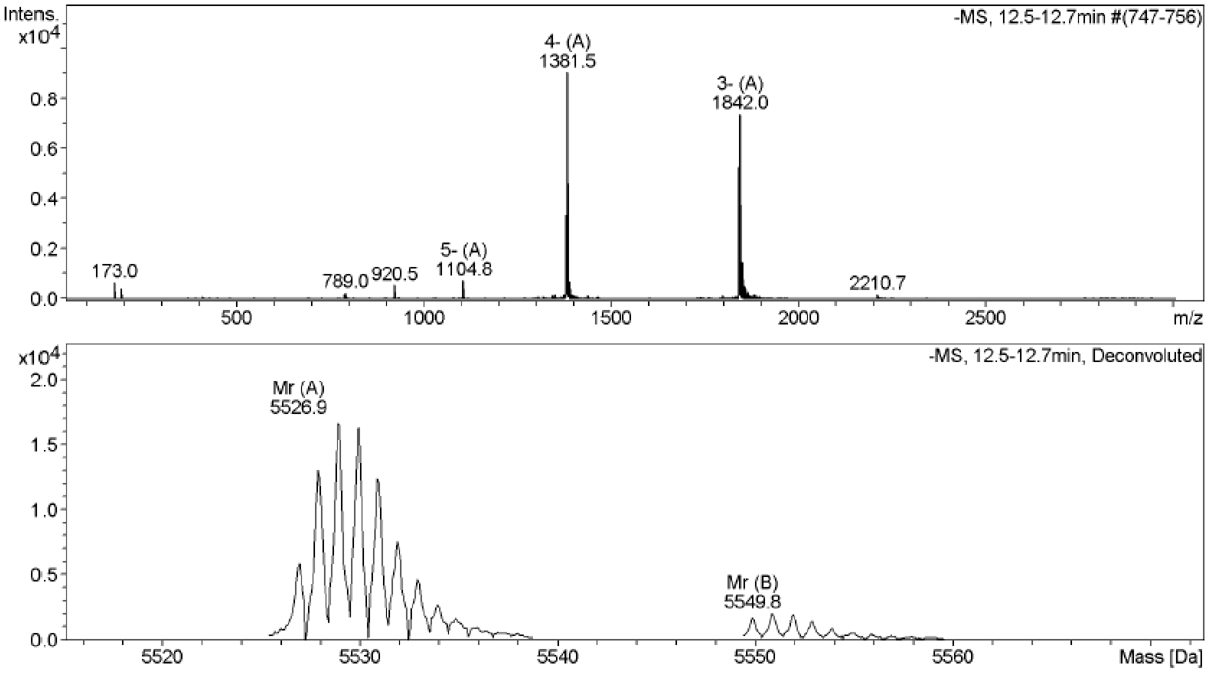

Figure S5: LC-MS analysis of conjugate **12**.

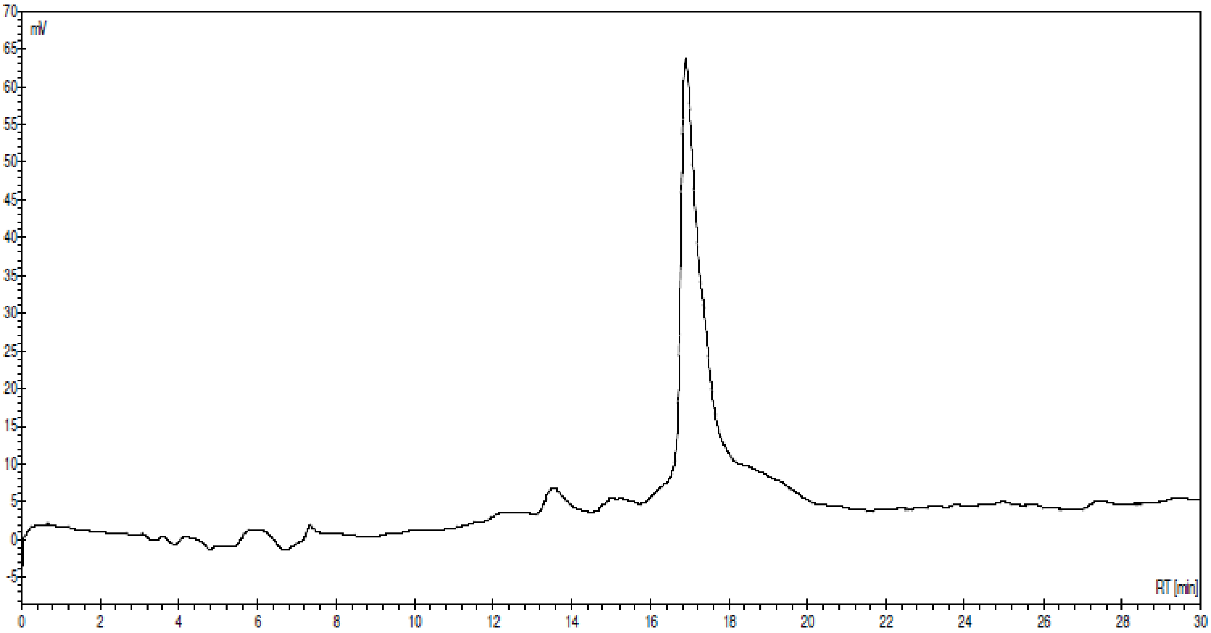

Figure S6: Chromatogram of conjugate **12** after purification.

Conjugate **13** calculated exact mass: 5447.7; found 5444.7; 5465.7 (+ Na^+).

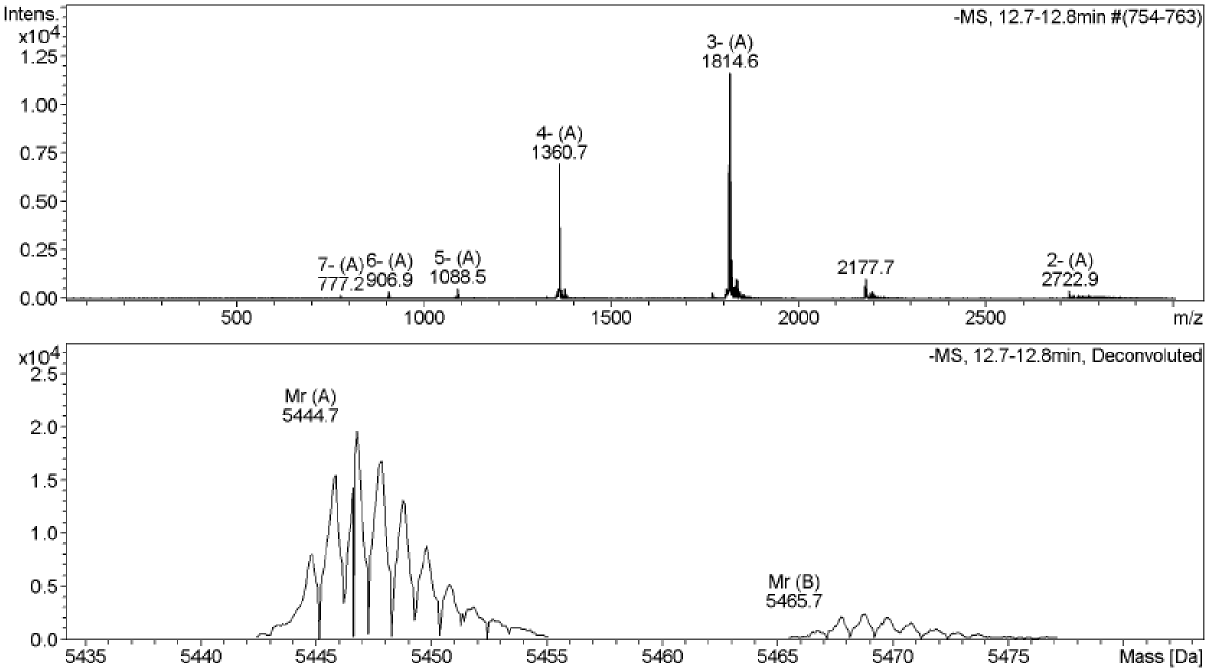

Figure S7: LC-MS analysis of conjugate **13**.

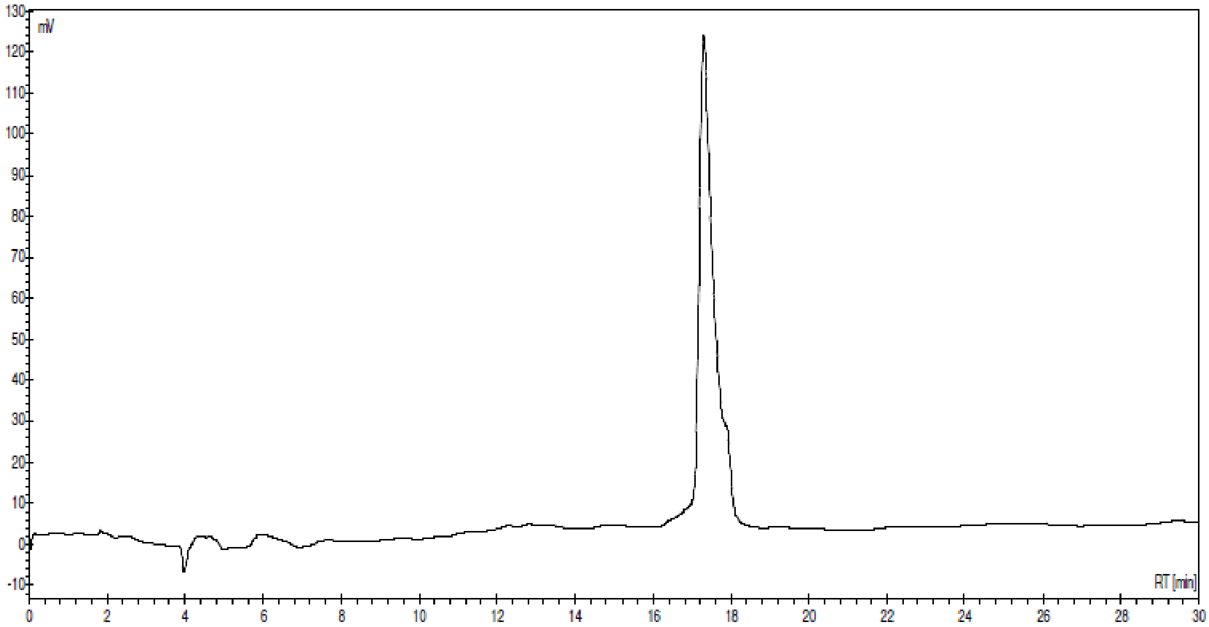

Figure S8: Chromatogram of conjugate **13** after purification.

Conjugate **16** calculated exact mass: 5431.3; found 5433.3.

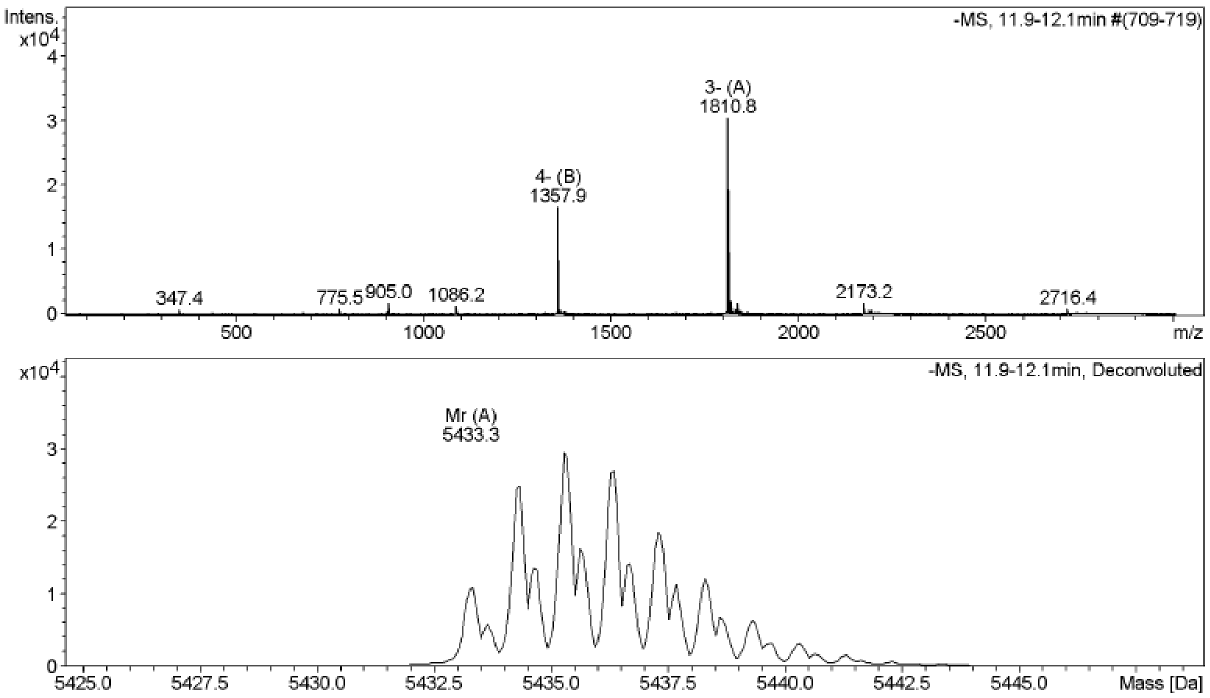

Figure S9: LC-MS analysis of conjugate **16**.

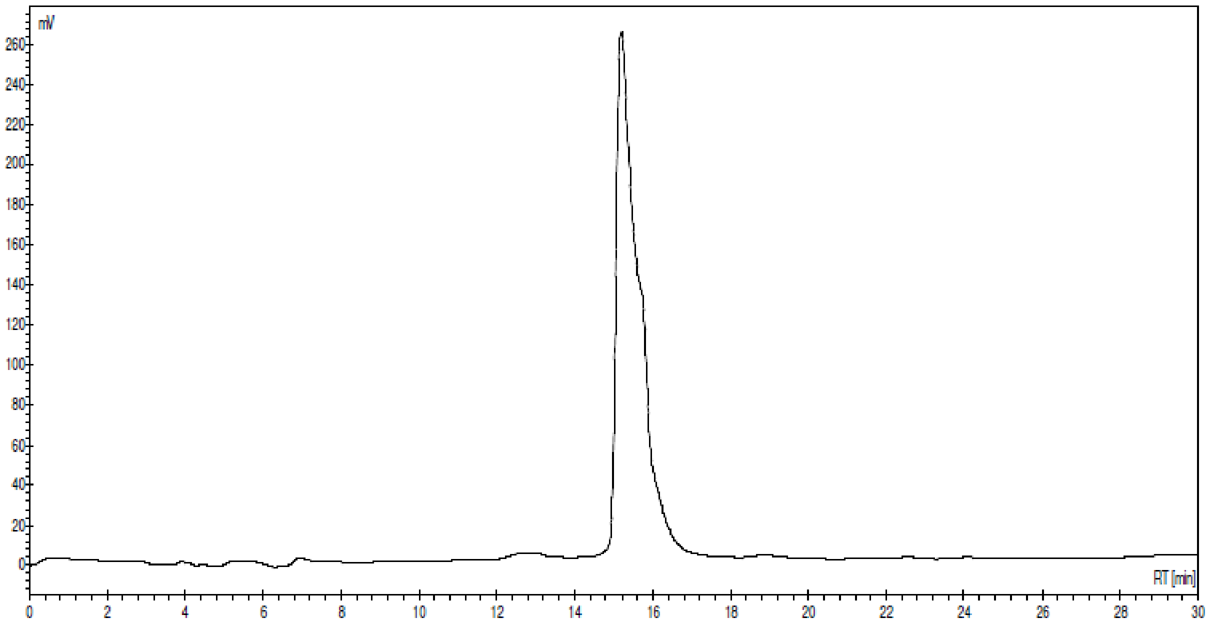

Figure S10: Chromatogram of conjugate **16** after purification.

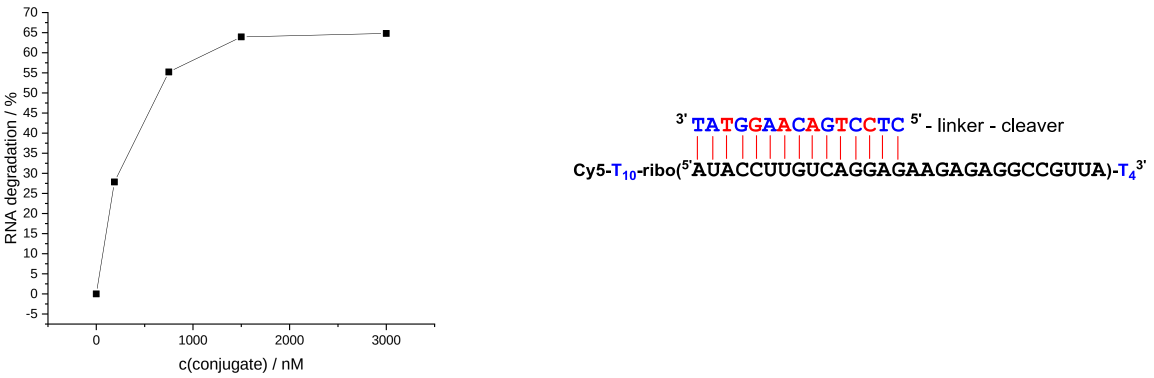

Figure S11: Conjugate **16**, used as a negative control (Figure 4), is an active cleaver of the complementary model RNA. Cleavage is shown as a function of conjugate concentration.

Preparation of RNA transcripts 14 and 15.

Sequence of 155-mer **14**:

5'-AGGGAUCCUGCUGUAUGAUAUGGUGUGGAGAUAUUCCUUUCGAGCAGCGA
UGCUUGAUACAGGAACAACAUUUACAACUCAUUCCAGAUCCCAGGCCCCUGGAG
GCUGCCUCCCAACAGUGGGGAAGAGUGACUCUCCAGGGGUCCUAGGUAC

Sequence of 412-mer **15**:

5'-GGUCCACCUCCACAGCCUGUCGCCGGGGCCCAGCAAAUAGCAGCCUUUCUGGC
AGGUCCUCCCCUCUCUUGUCAGAUGCCCGAGGGAGGGGAAGCUUCUGUCUCCAG
CUUCCCGAGUACCAGUGACACGUCUCGCCAAGCAGGACAGUGCUUGAUACAGGA
ACAACAUUUACAACUCAUUCCAGAUCCCAGGCCCCUGGAGGCUGCCUCCCAACAG
UGGGGAAGAGUGACUCUCCAGGGGUCCUAGGCCUCAACUCCUCCCAUAGAUACU
CUCUUCUUCUCAUAGGUGUCCAGCAUUGCUGGACGAUAUCGGAUCCCGGGCCCG
UCGACUGCAGAGGCCUGCAUGCAAGCUUGGCGUAAUCAUGGUCAUAGCUGUUUC
CUGUGUGAAAUUGUUAUCCGCUCACAAUUCCACA

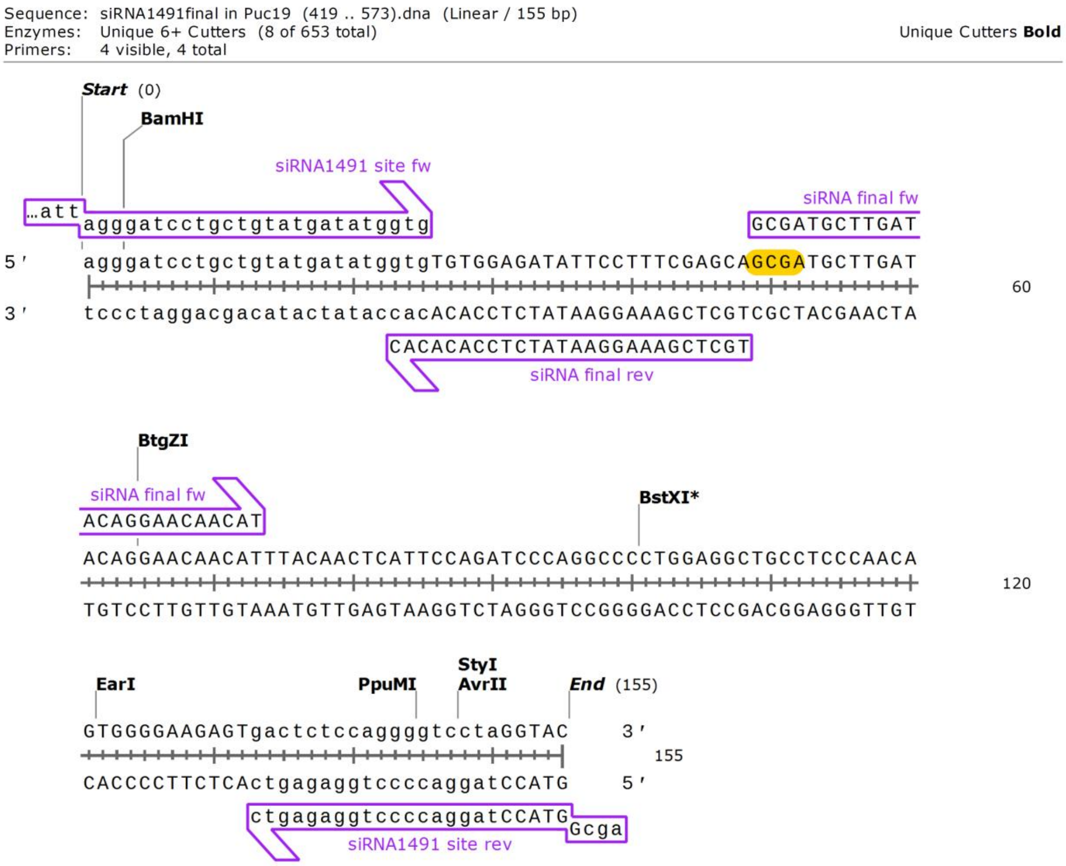

Figure S12: PCR strategies for construction of the T7 transcription template of 155-mer RNA substrate. The used PCR primers are marked in magenta and the inserted 5'-GCGA tetraloop sequence is highlighted in ochre.

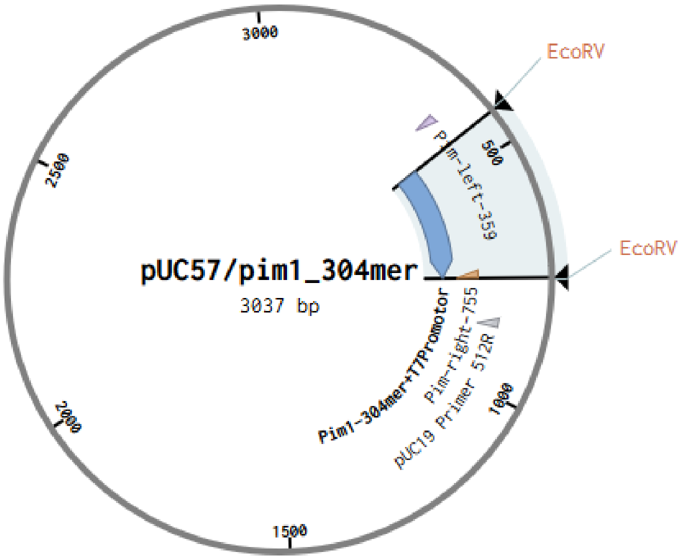

Figure S13: Map of pUC57 vector and primer orientation.

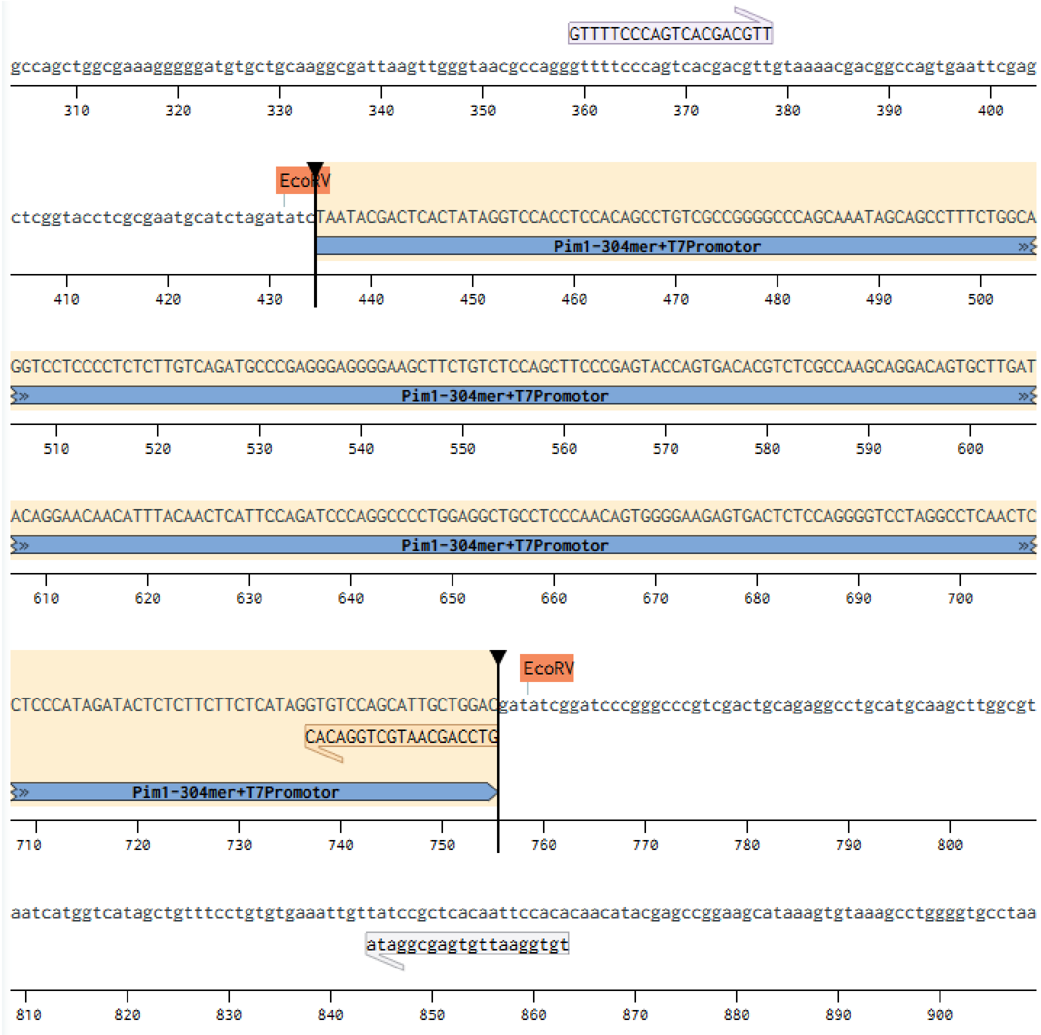

Figure S14: Positioning of the two primer pairs on the pUC57 vector [3].

Cleavage assay with unlabeled 412-mer **15**.

GMP-primed 412-mer **15** (250 nM) was incubated in a final volume of 10 µL with 1 µM conjugate in presence of 5 mg/ml poly(vinylsulfuric acid) [4] in either 50 mM Na_2HPO_4, 50 mM Tris-HCl pH 8.0 buffer or in 50 mM Na_2HPO_4 pH 8.0 at 37 °C for 1 – 24 h. Prior to electrophoresis 10 µL gel loading buffer (8 M urea, 20 mM EDTA, 0.2 % crocein orange G) was added to each sample. The gel (8 % denaturing PAGE) was subsequently stained with SYBR Gold (Thermo Fisher Scientific) and photographed using a Fusion FX (Vilber Lourmat). Digital images were integrated by means of the Phoretix 1D Quantifier software (Totalllab) assuming that the staining intensity increases linearly with the fragment length.

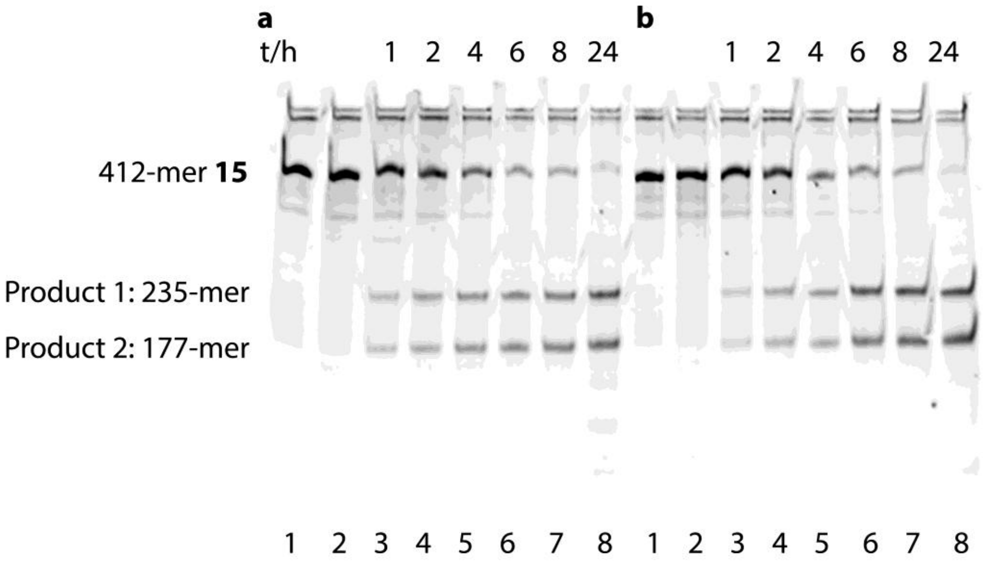

Figure S15: Cleavage RNA **15** (250 nM) by conjugate **12** (1 µM). When incubated in **a**) 50 mM Na_2HPO_4, 50 mM Tris-HCl pH 8.0 buffer or in **b**) 50 mM Na_2HPO_4 pH 8.0 with conjugate **12** (1 µM), two fragments became visible in gel electrophoresis after staining with SYBR Gold. Cleavage reactions (10 µL) were stopped by adding 10 µL of RNA loading buffer (8 M urea, 20 mM EDTA, 0.2 % crocein orange) after the indicated times (lanes a3-a8, b3-b8). Control reactions were carried out in absence of conjugate **12** for 0 h (lane a1, b1) and 24 h (a2, b2).

Secondary structure predictions.

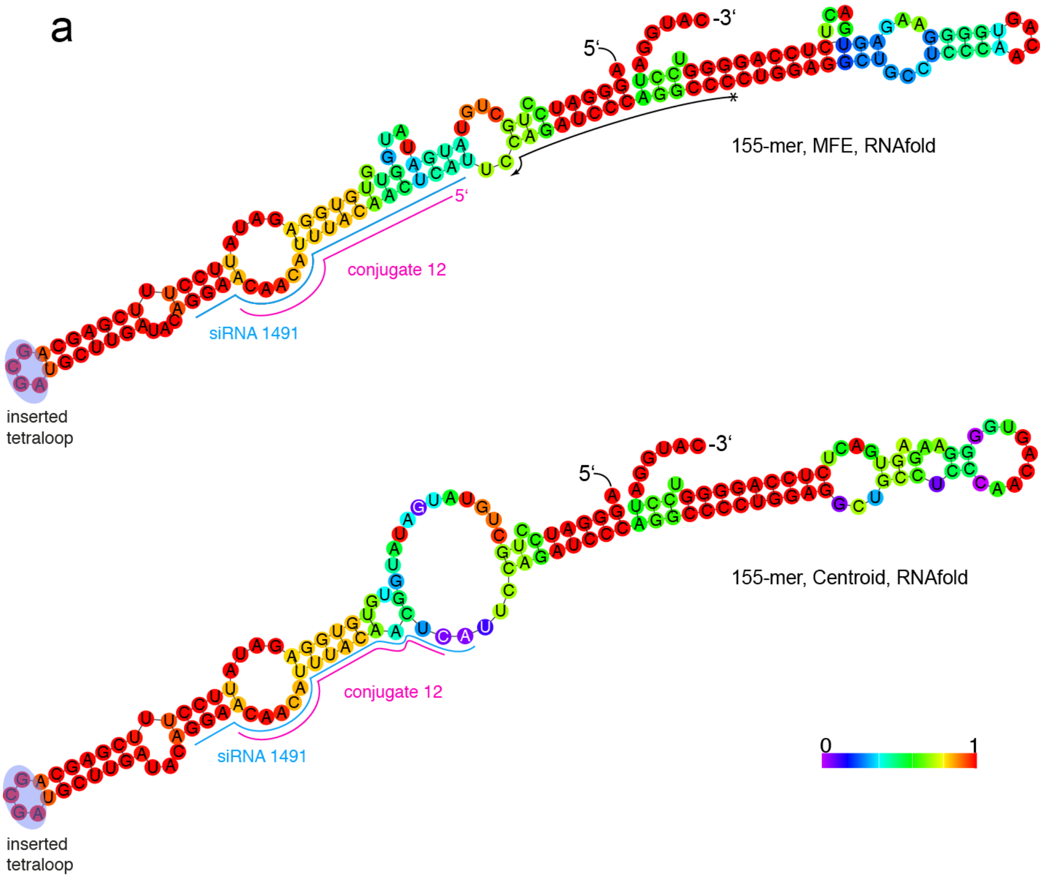

b

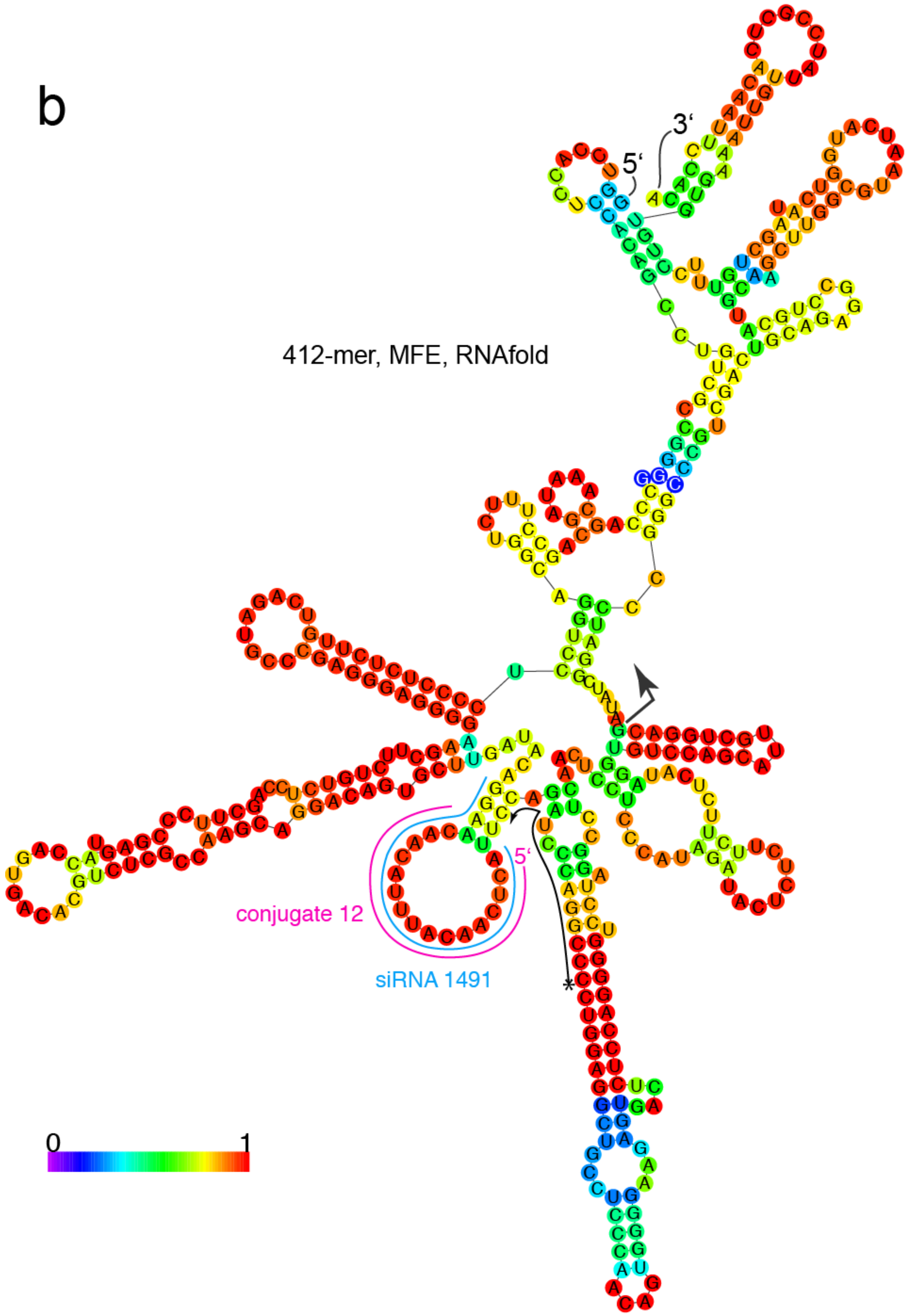

c

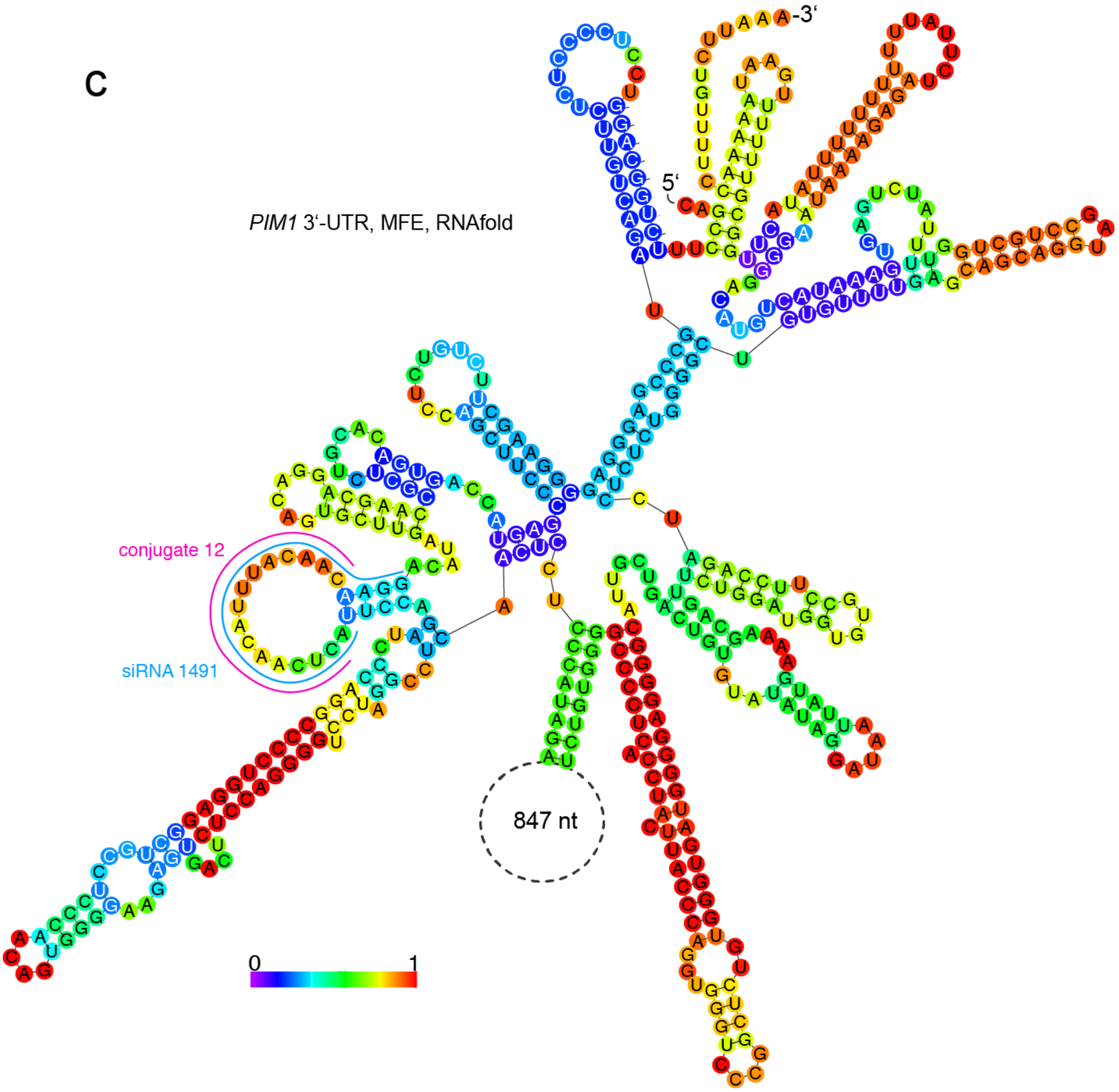

Figure S16: RNA secondary structure predictions of the (**a**) 155-mer and (**b**) the 412-mer representing portions of the 3'-UTR of the mRNA encoding the human oncogenic kinase PIM1, in comparison with (**c**) the structure prediction of the entire *PIM1* 3'-UTR (the internal portion of 847 nucleotides omitted for the sake of clearness). 2D structures were predicted using RNAfold with default settings. The centroid structure prediction is only shown for the 155-mer, but centroid structures for the 412-mer and the entire 3'-UTR are very similar to the corresponding MFE structures. The color code indicates the probability (red: highest; blue: lowest) that a nucleotide is involved in base pairing or is unpaired. The target sites of the validated siRNA 1491 and that of conjugate **12** are marked by the light blue and pink lines, respectively, in panels **a** to **c**. In panels **a** and **b**, the binding site of the reverse transcriptase primer 5′-GGGCCTGGGATCTGG-3′ is shown as a black line with arrowhead at its 3'-end and asterisk at the 5'-end to indicate its 5'-^{32}P-end label. In the 412-mer structure in panel **b**, the large black arrow indicates the 3'-proximal nucleotides derived from plasmid pUC57 (see text above).

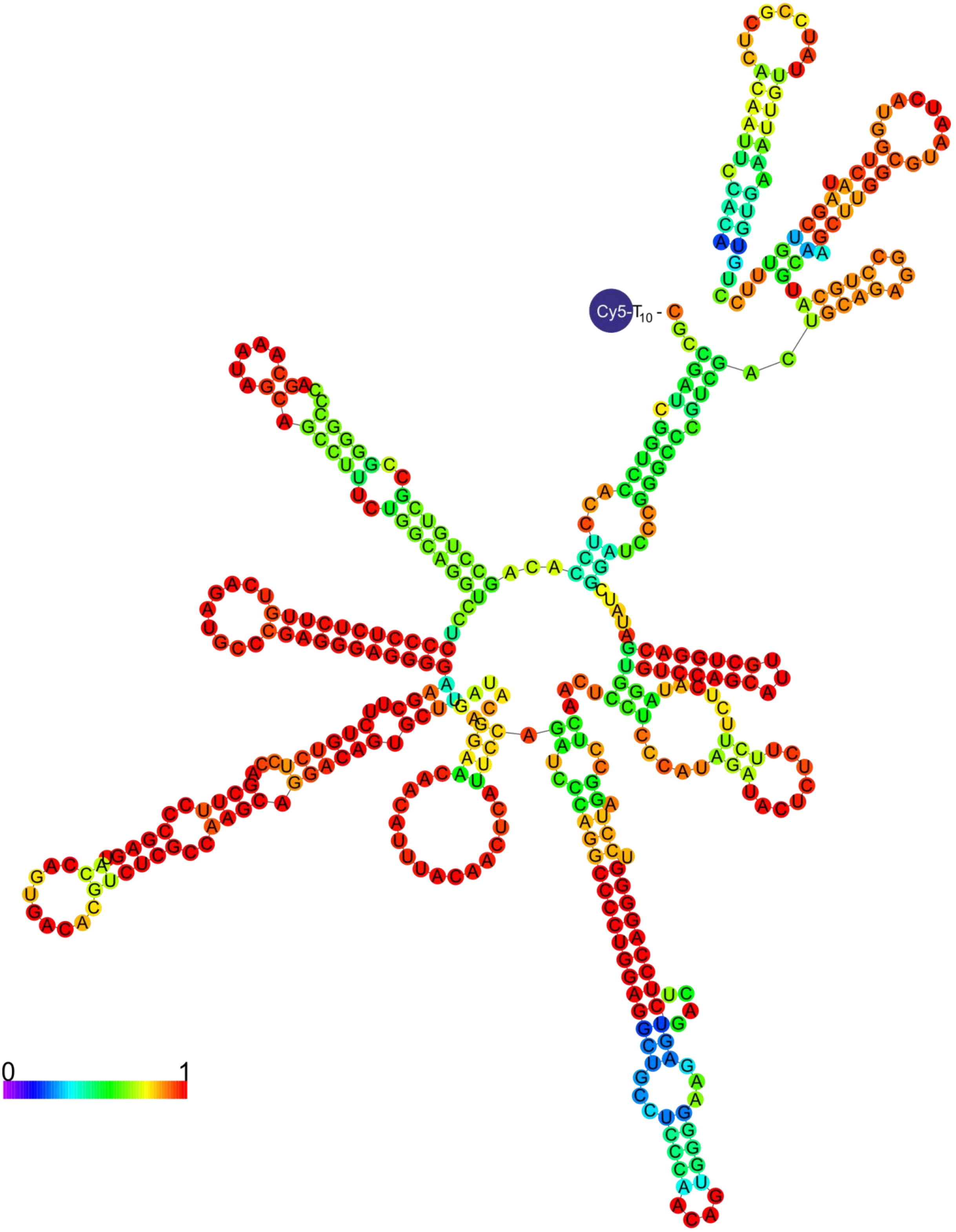

Figure S17: The Cy5-labeled 430-mer **17**, a derivative of the 412-mer, was extended at the 5'-end by ten deoxythymidine residues and eight RNA residues (5'-Cy5-TTT TTT TTT T CGCCGAUC) relative to the 412-mer.

References

1. Gnaccarini, C.; Peter, S.; Scheffer, U.; Vonhoff, S.; Klussmann, S.; Göbel, M.W. Site-specific cleavage of RNA by a metal-free artificial nuclease attached to antisense oligonucleotides. *J. Am. Chem. Soc*. **2006**, *128*, 8063–8067.
2. Nanodrop: Gray, D. M.; Hung, S. H.; Johnson, K. H. Absorption and Circular-Dichroism Spectroscopy of Nucleic-Acid Duplexes and Triplexes. *Methods in Enzymology* **1995**, *246*, 19.
3. Benchling [Biology Software]. (2018). Retrieved from https://benchling.com.
4. Smith, B. D.; Soellner, M. B.; Raines, R. T. Potent Inhibition of Ribonuclease A by Oligo(vinylsulfonic Acid). *J. Biol. Chem.* **2003**, *23*, 20934–20938.
5. ViennaRNA Web Services. Available online: http://rna.tbi.univie.ac.at/ (accessed on 22.11. 2018)

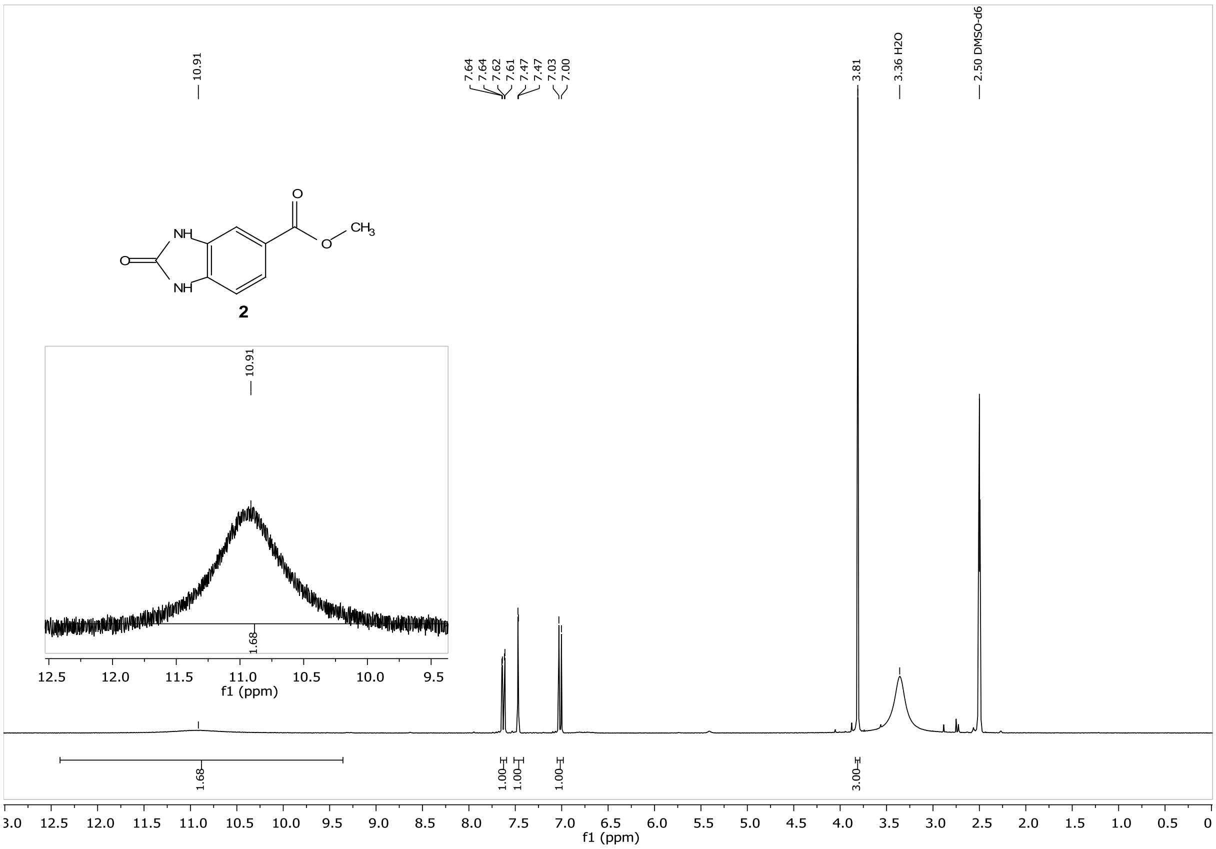
10.91
7.64
7.64
7.62
7.61
7.47
7.47
7.03
7.00
3.81
3.36 H2O
2.50 DMSO-d6
NH
NH
O
O
O
CH3
2
1.68
1.00
1.00
1.00
3.00
f1 (ppm)

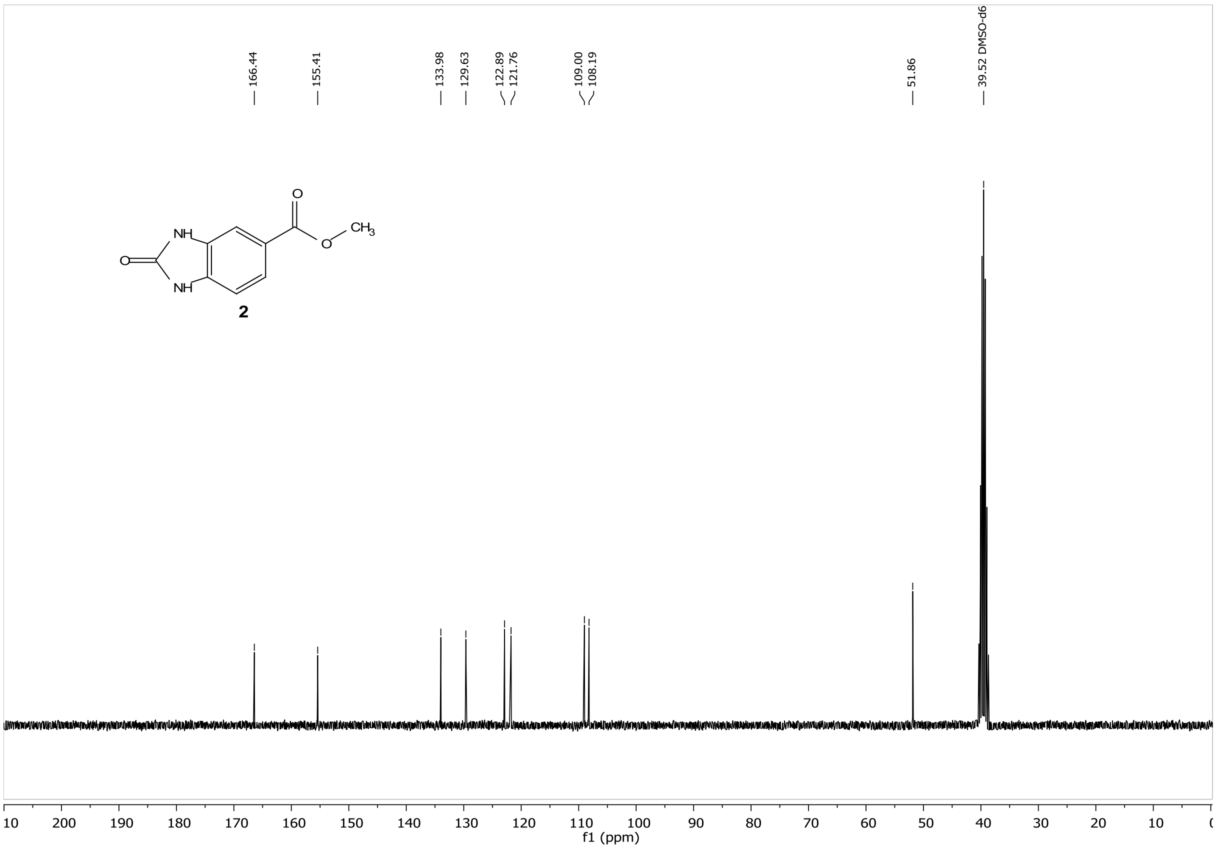
166.44
155.41
133.98
129.63
122.89
121.76
109.00
108.19
51.86
39.52 DMSO-d6
NH
NH
O
O
O
CH3
2
f1 (ppm)

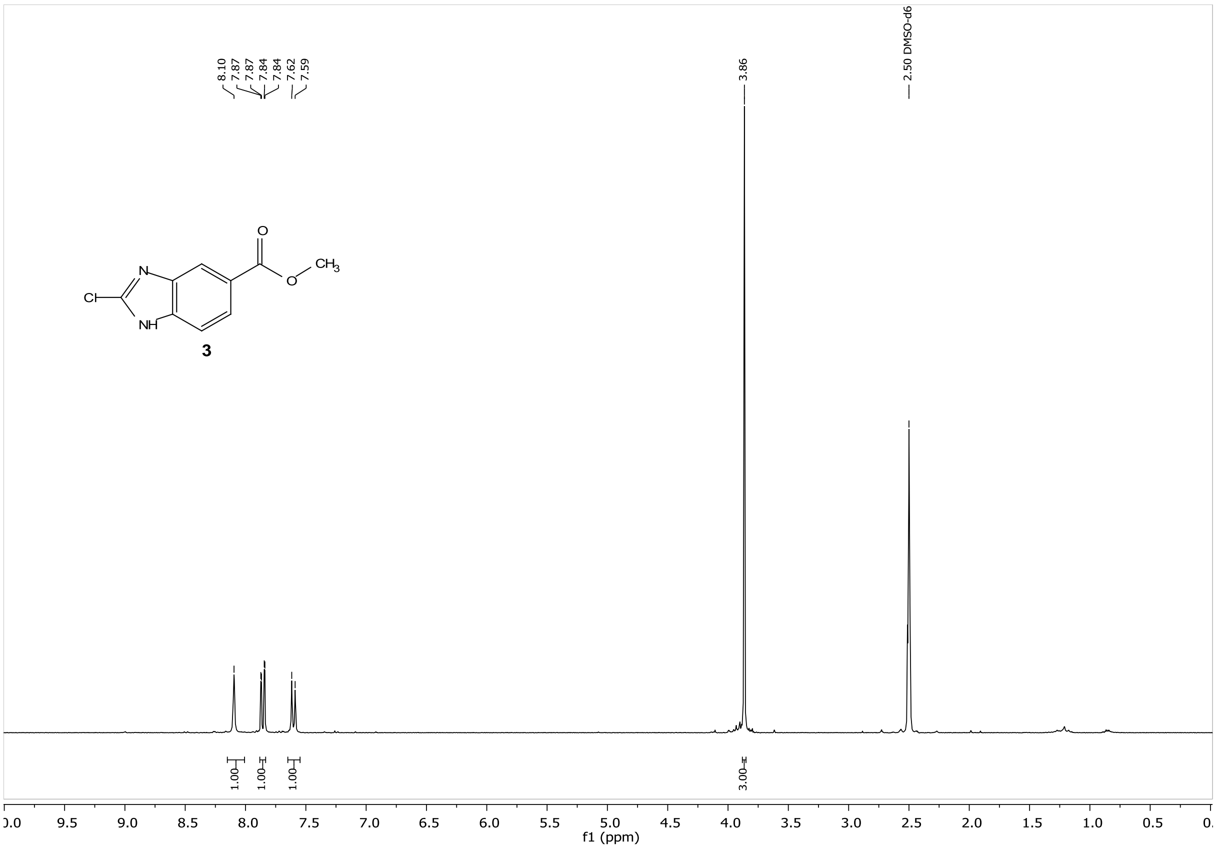
8.10
7.87
7.87
7.84
7.84
7.62
7.59
3.86
2.50 DMSO-d6
Cl
N
NH
O
O
CH3
3
1.00
1.00
1.00
3.00
f1 (ppm)

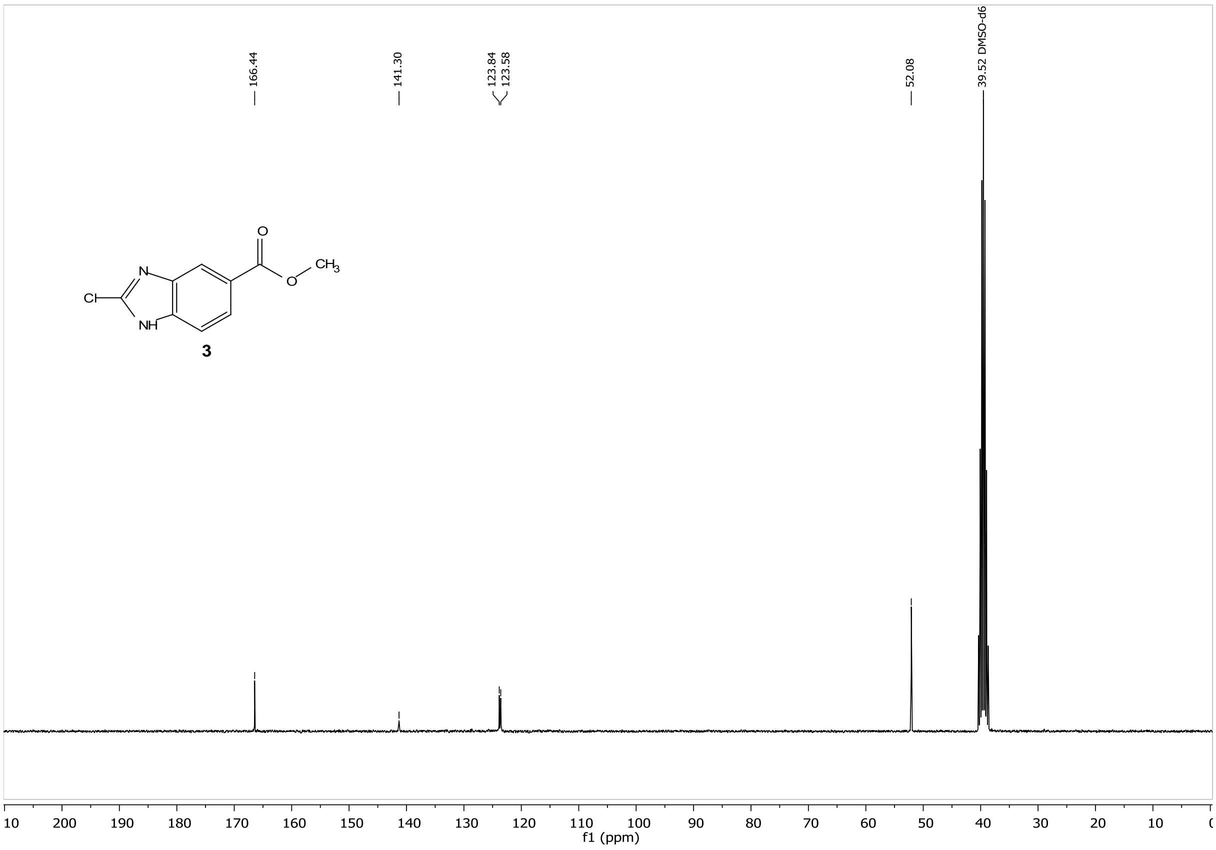
166.44
141.30
123.84
123.58
52.08
39.52 DMSO-d6
Cl
N
NH
O
O
CH3
3
f1 (ppm)

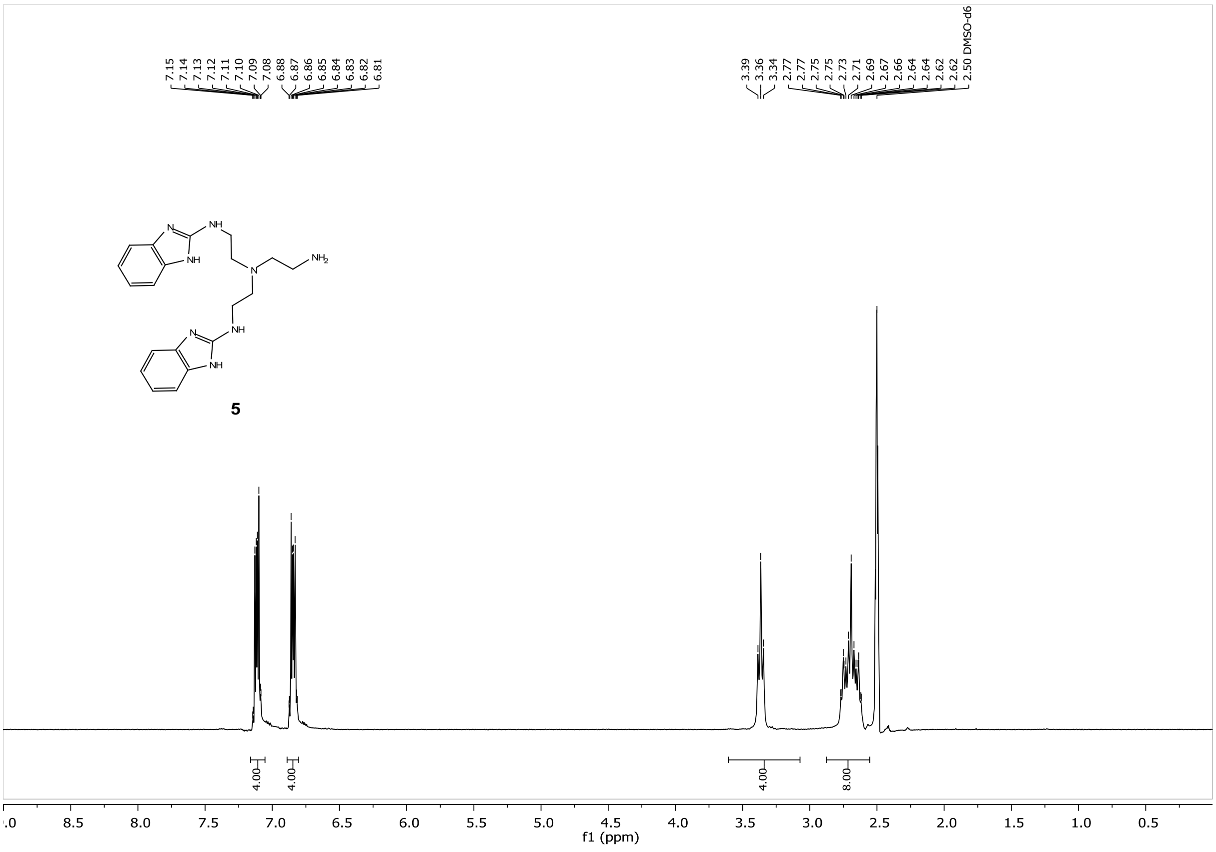
7.15
7.14
7.13
7.12
7.11
7.10
7.09
7.08
6.88
6.87
6.86
6.85
6.84
6.83
6.82
6.81
3.39
3.36
3.34
2.77
2.77
2.75
2.75
2.73
2.71
2.69
2.67
2.66
2.64
2.64
2.62
2.62
2.50 DMSO-d6
NH
N
NH
N
NH₂
NH
N
NH
5
4.00
4.00
4.00
8.00
f1 (ppm)

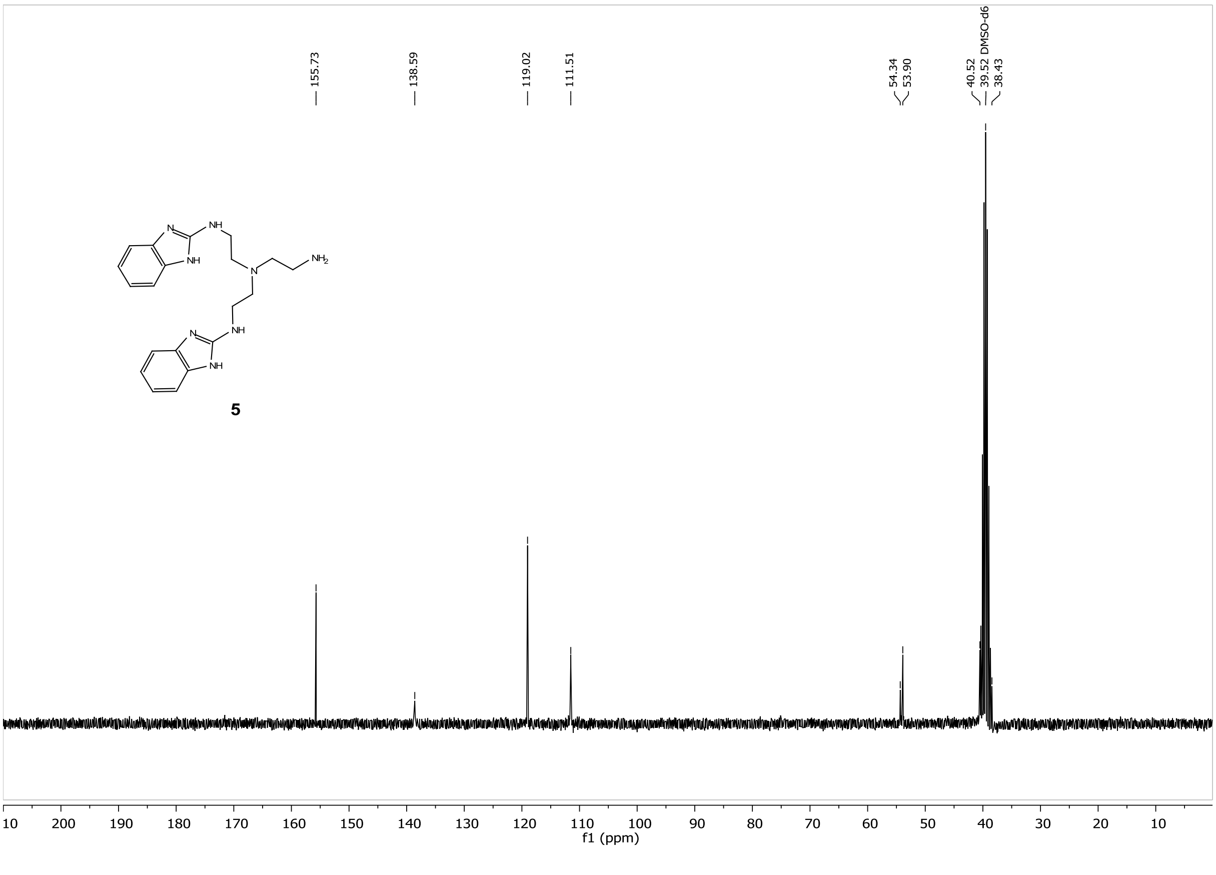
155.73
138.59
119.02
111.51
54.34
53.90
40.52
39.52 DMSO-d6
38.43
5
f1 (ppm)

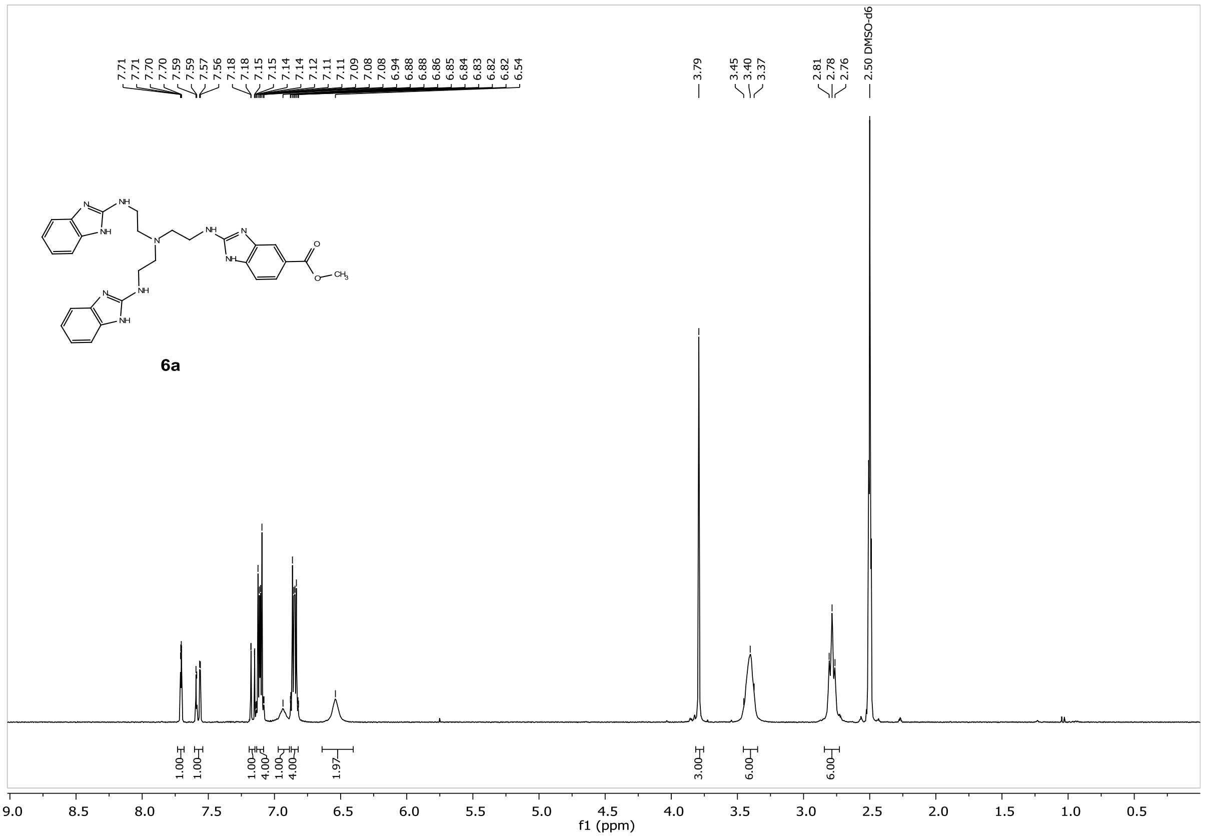
7.71 7.71 7.70 7.70 7.59 7.59 7.57 7.56 7.18 7.18 7.15 7.15 7.14 7.14 7.12 7.11 7.11 7.09 7.08 7.08 6.94 6.88 6.88 6.86 6.85 6.84 6.83 6.82 6.82 6.54
3.79
3.45 3.40 3.37
2.81 2.78 2.76
2.50 DMSO-d6
6a
1.00 1.00 1.00 4.00 1.00 4.00 1.97 3.00 6.00 6.00
f1 (ppm)

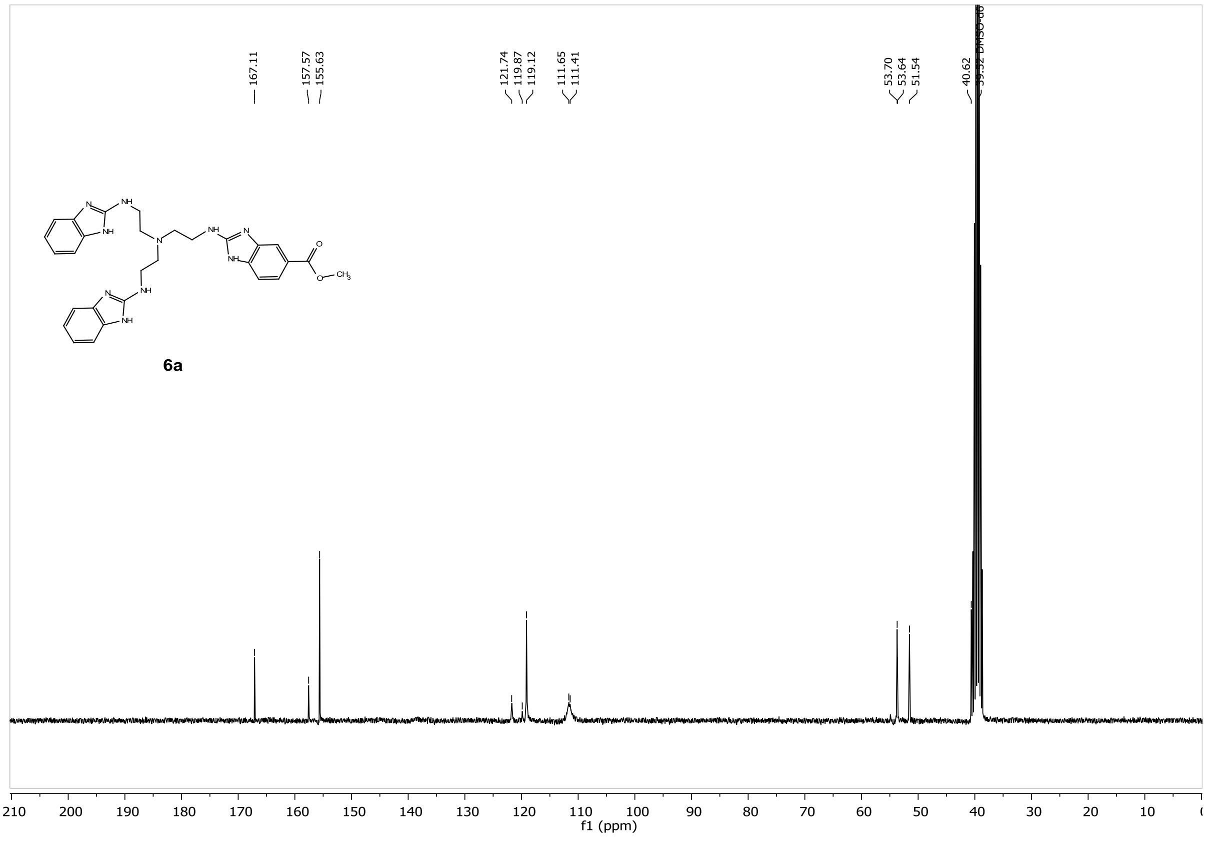
167.11
157.57 155.63
121.74 119.87 119.12
111.65 111.41
53.70 53.64 51.54
40.62 39.52 DMSO-d6
6a
f1 (ppm)

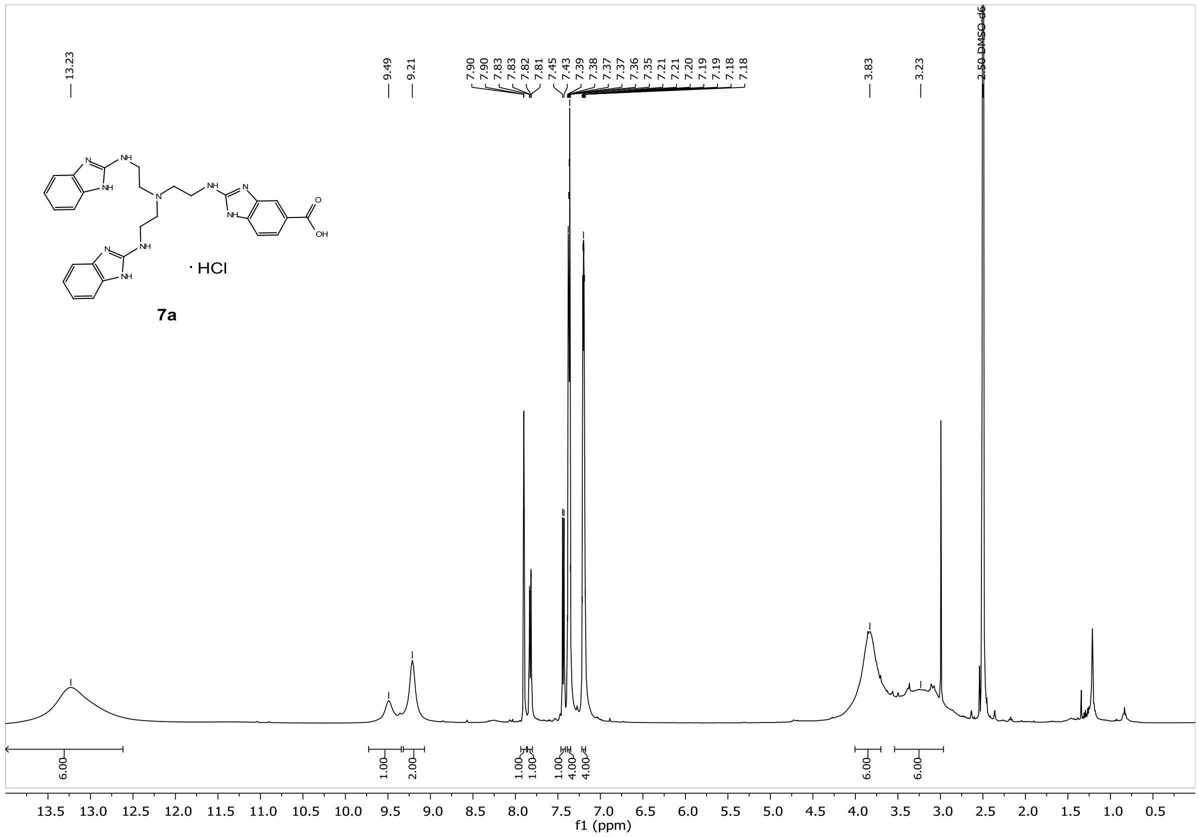
13.23
9.49
9.21
7.90
7.90
7.83
7.83
7.82
7.81
7.45
7.43
7.39
7.38
7.37
7.37
7.36
7.35
7.21
7.21
7.20
7.19
7.19
7.18
7.18
3.83
3.23
2.50 DMSO-d6
· HCl
7a
6.00
1.00
2.00
1.00
1.00
1.00
4.00
4.00
6.00
6.00
13.5 13.0 12.5 12.0 11.5 11.0 10.5 10.0 9.5 9.0 8.5 8.0 7.5 7.0 6.5 6.0 5.5 5.0 4.5 4.0 3.5 3.0 2.5 2.0 1.5 1.0 0.5
f1 (ppm)

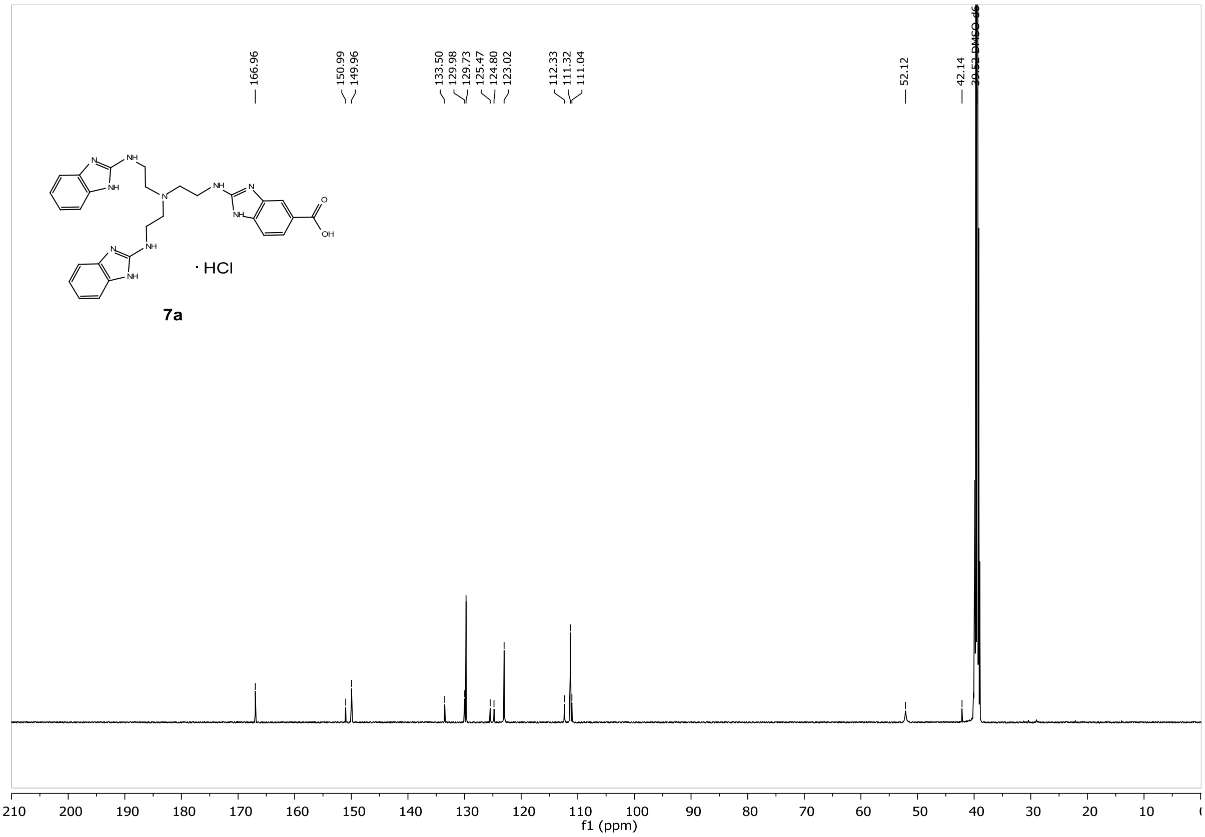
166.96
150.99
149.96
133.50
129.98
129.73
125.47
124.80
123.02
112.33
111.32
111.04
52.12
42.14
39.52 DMSO-d6
· HCl
7a
210 200 190 180 170 160 150 140 130 120 110 100 90 80 70 60 50 40 30 20 10
f1 (ppm)

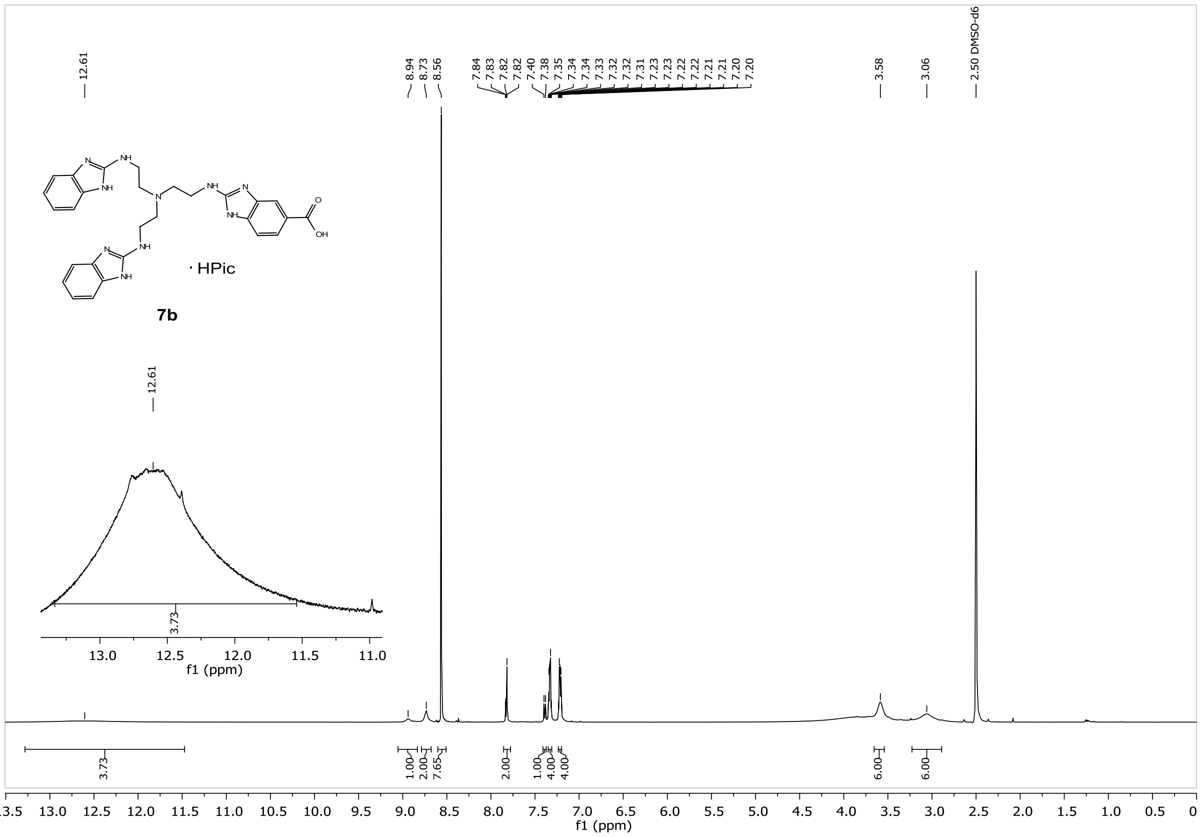
12.61
8.94
8.73
8.56
7.84
7.83
7.82
7.82
7.40
7.38
7.35
7.34
7.34
7.33
7.32
7.32
7.31
7.23
7.23
7.22
7.22
7.22
7.21
7.21
7.20
7.20
3.58
3.06
2.50 DMSO-d6
· HPic
7b
f1 (ppm)

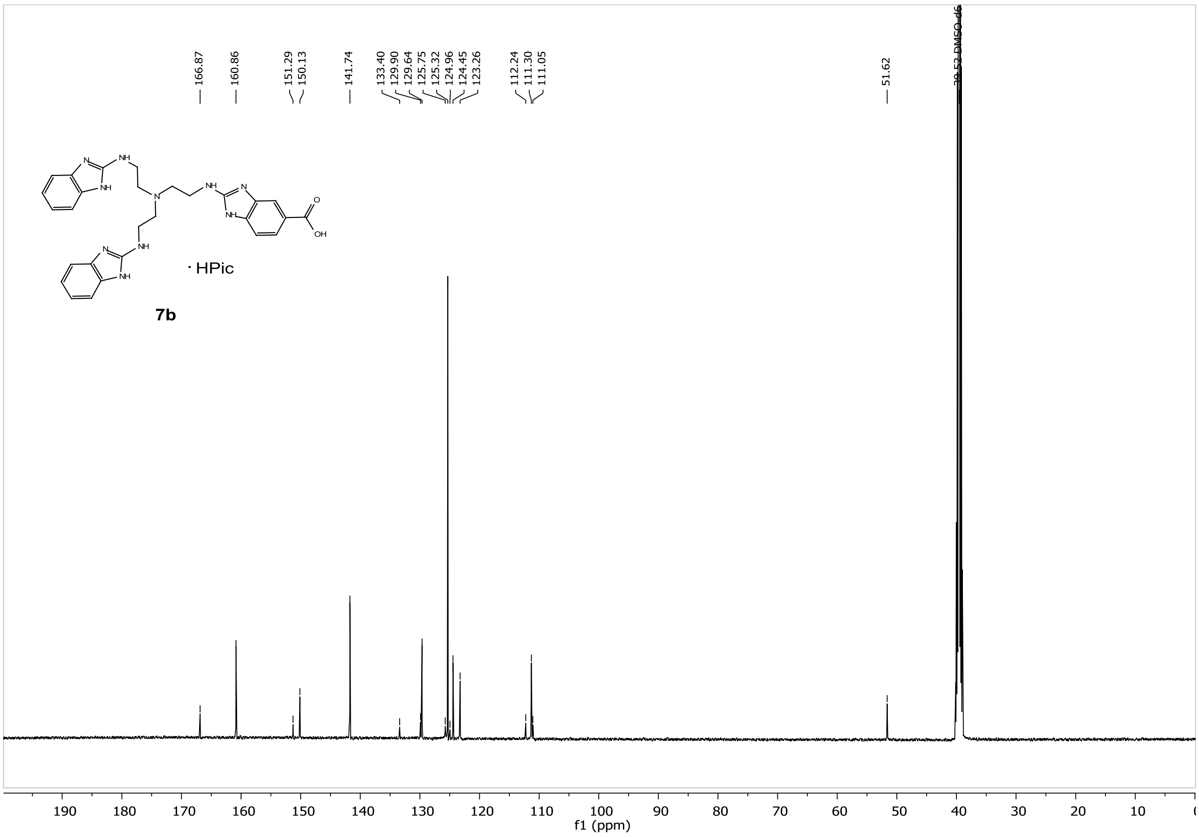
166.87
160.86
151.29
150.13
141.74
133.40
129.90
129.64
125.75
125.32
124.96
124.45
123.26
112.24
111.30
111.05
51.62
39.52 DMSO-d6
· HPic
7b
f1 (ppm)
